# Sexual Dimorphism in Lipid Metabolism and Gut Microbiota in Mice Fed a High-Fat Diet

**DOI:** 10.3390/nu15092175

**Published:** 2023-05-02

**Authors:** Qi Zhu, Nathan Qi, Ling Shen, Chunmin C. Lo, Meifeng Xu, Qing Duan, Nicholas J. Ollberding, Zhe Wu, David Y. Hui, Patrick Tso, Min Liu

**Affiliations:** 1 Department of Pathology and Laboratory Medicine, University of Cincinnati College of Medicine, Cincinnati, OH 45237, USA; zhuqu@ucmail.uc.edu (Q.Z.);; 2 Department of Molecular and Integrative Physiology, University of Michigan, Ann Arbor, MI 48109, USA; nathanqi@med.umich.edu (N.Q.);; 3 Department of Biomedical Sciences, Diabetes Institute, Heritage College of Osteopathic Medicine, Ohio University, Athens, OH 45701, USA; 4 Division of Biostatistics and Epidemiology, Cincinnati Children’s Hospital Medical Center, Department of Pediatrics, University of Cincinnati College of Medicine, Cincinnati, OH 45237, USA

**Keywords:** sexual dimorphism, gut microbiota, dyslipidemia, *Akkermansia*, apolipoproteins

## Abstract

The gut microbiome plays an essential role in regulating lipid metabolism. However, little is known about how gut microbiome modulates sex differences in lipid metabolism. The present study aims to determine whether gut microbiota modulates sexual dimorphism of lipid metabolism in mice fed a high-fat diet (HFD). Conventional and germ-free male and female mice were fed an HFD for four weeks, and lipid absorption, plasma lipid profiles, and apolipoprotein levels were then evaluated. The gut microbiota was analyzed by 16S rRNA gene sequencing. After 4-week HFD consumption, the females exhibited less body weight gain and body fat composition and significantly lower triglyceride levels in very-low-density lipoprotein (VLDL) and cholesterol levels in high-density lipoprotein (HDL) compared to male mice. The fecal microbiota analysis revealed that the male mice were associated with reduced gut microbial diversity. The female mice had considerably different microbiota composition compared to males, e.g., enriched growth of beneficial microbes (e.g., *Akkermansia*) and depleted growth of *Adlercreutzia* and *Enterococcus*. Correlation analyses suggested that the different compositions of the gut microbiota were associated with sexual dimorphism in body weight, fat mass, and lipid metabolism in mice fed an HFD. Our findings demonstrated significant sex differences in lipid metabolism and the microbiota composition at baseline (during LFD), along with sex-dependent responses to HFD. A comprehensive understanding of sexual dimorphism in lipid metabolism modulated by microbiota will help to develop more sex-specific effective treatment options for dyslipidemia and metabolic disorders in females.

## 1. Introduction

The gut microbiota is a complex microbial community comprising trillions of complex microorganisms, profoundly affecting energy and lipid metabolism [1]. The microbiota interacts symbiotically with its host and contributes to certain critical metabolic functions, e.g., facilitating the digestion and absorption of macro- and micronutrients from food [2]. The intestinal epithelium is one of the most significant barriers between the outside environment and the host, forming a selective barrier that permits the absorption of macronutrients while maintaining an effective defense against bacteria and other toxins. The gut microbiota regulates intestinal permeability and mucosal mast cell activation [3]. Many studies have shown that dyslipidemia is associated with altered gut microbiota composition [1]. Therefore, modulating gut microbiota may be a beneficial strategy to prevent the development of dyslipidemia [4].

Various factors, including host genetics, diet, and gonadal hormones, can alter the gut microbiome [5,6]. Sex differences in gut microbiota have been reported in humans and rodents [6,7,8,9]. However, little is known about how gut microbiome modulates sex differences in lipid metabolism. Since men and women differ in energy intake and metabolism, we hypothesize that sex-specific microbiome signatures may play an important role in the difference in lipid absorption and metabolism between male and female mice. To test these hypotheses, we determined the responses of male and female germ-free (GF) and conventional (CV) mice to an HFD in certain metabolic aspects, including body weight and fat composition, lipid absorption, plasma lipid profile, lipoprotein distribution, and circulating apolipoprotein levels. The effects of sex on the association of HFD and microbiota composition were evaluated by sequencing the V4 hypervariable region of the 16S rRNA gene from fecal samples collected in CV mice fed LFD and HFD. Overall, our data revealed significant differences in the composition of gut microbiota between male and female mice. Furthermore, we demonstrated that the sex-dependent microbial response to HFD was correlated to body weight, fat mass, and lipid metabolic parameters. These findings help us to better understand the gender-specific changes in lipid metabolism in response to HFD.

## 2. Materials and Methods

### 2.1. Animals

All animal experiments were performed at the University of Michigan Animal Phenotyping Core in accordance with the recommendations put forth in the Guide for the Care and Use of Laboratory Animals and were approved by the University of Michigan Institutional Animal Care and Use Committee. Adult male and female GF mice (C57Bl/6NCrl) were derived and maintained at the germ-free mouse facility at the University of Michigan. The CV mice (C57BL/6J) were purchased from The Jackson Laboratory (Bar Harbor, ME, USA). These animals (12–13 weeks old) were housed in a room under a 12 h:12 h light–dark cycle and temperature (22  ±  1 °C)- and humidity (40–60%)-controlled environment. All animals had ad libitum access to food and water.

### 2.2. Materials

A semi-synthetic diet containing 5% sucrose polybehenate, a non-absorbable food additive, was prepared by Research Diets, Inc. (New Brunswick, NJ, USA), as we reported previously [10]. Additionally, two pelleted semi-purified, nutritionally complete experimental diets (AIN-93M [11]) were purchased from Research Diets, Inc., New Brunswick, NJ, USA. The high-fat diet (HFD, catalog #: D03082706) contained 20% fat (19 g of butter oil and 1 g of soybean oil in 100 g of diet to provide essential fatty acids), and the low-fat diet (LFD, catalog #: D03082705) contained 3 g of butter oil and 1 g of soybean oil in 100 g of diet [12]. We equalized the amount of protein and all essential minerals and vitamins required for rodents [8] per kJ for both HFD and LFD [12]. Diets given to the GF mice were sterilized with double gamma irradiation, and the diets given to CV mice were regularly irradiated.

### 2.3. Experiments

A total of 28 GF mice (14 males and 14 females) and 28 CV mice (14 males and 14 females) were used in the present studies. To compare the fat absorption efficiency between male and female mice, mice were individually housed and fed the test diet containing 5% sucrose polybehenate for 5 days. After the 3rd day, the mouse cages were changed daily. Fecal samples were collected from each cage on the 4th and 5th days and stored at −80 °C until analysis for intestinal fat absorption by gas chromatography, as described below.

The mice were then switched to regular diet for two weeks to wash out the test diet above, the male and female GF and CV mice were randomly divided into two groups (total 8 groups). One group was fed LFD, and the other was provided HFD for 4 weeks. Body weight was recorded weekly. Submandibular blood samples were collected weekly using 25G needles from 5 h fasted mice (9 a.m.–5 p.m.) to determine the lipid profile, as described below. At the end of the 4-week feeding period, animal body composition was determined by the EchoMRI (Echo Medical Systems, 4 in 1–500, Houston, TX, USA). The animals were then anesthetized, and whole blood was collected by cardiac puncture. After centrifugation, plasma was collected and used for fast-performance liquid chromatography (FPLC) analyses of lipoprotein and apolipoprotein composition, as described below. Plasma apolipoproteins, including APOA1, APOA4, APOB48, and APOE, were analyzed by Western blot analyses. Fresh fecal pellets (at least 20 mg/mouse) were collected directly from male and female CV mice before (Week 0) and after LFD or HFD feeding for 4 weeks (Week 4) and stored at −80 °C for the analyses of microbial community composition by 16S rRNA gene sequencing at the University of Michigan Microbiome Core.

### 2.4. Gas Chromatography (GC) Assay for Fat Absorption

Fat absorption in mice was determined by the sucrose polybehenate method, as we described previously [10]. Briefly, the weighed fecal pellet samples (~10 mg) were saponified, methylated, and extracted. Methyl esters of fatty acids were analyzed by GC, the Shimadzu GC-2014 equipped with an auto-injector (Shimadzu Scientific Instruments Inc., Columbia, MD, USA). The GC conditions were column temperature ramping by holding at 120 °C for one minute followed by an increase of 5 °C/min from 120 to 240 °C. The temperature of the injector and flame ionization detector was 250 °C. The carrier gas was helium, with a column flow rate of 2.5 mL/min. Fatty acid identification was determined using retention times of authenticated fatty acid methyl ester standards (Matreya LLC Inc., Pleasant Gap, PA, USA). Fatty acid composition data were expressed as weight percent of total fatty acids (mg fatty acid/100 mg total fatty acids). Since behenic acid is entirely excreted when given as sucrose polybehenate, fat absorption was calculated from the ratios of behenic acid to other fatty acids in the diet and in the feces as analyzed by fatty acid methyl esters.

### 2.5. Biochemical Analysis

Plasma triglyceride (TG) was measured using a kit from Randox Laboratories (Kearneysville, WV, USA), and PL was measured using a kit from Wako Diagnostics (Mountain View, CA, USA). Total cholesterol (CHOL) was measured using Infinity CHOL Liquid Stable Reagent (Fisher Diagnostics, Thermo Scientific, Middletown, VA, USA), and NEFA was measured using the kits from FUJIFILM Medical Systems USA (Miami, FL, USA). All assays were performed according to the manufacturer’s protocols.

### 2.6. Fast-Performance Liquid Chromatography (FPLC) for Lipoprotein Fractions

Lipoprotein fractions were collected using a Superose 6 10/300 GL column in the ÄKTA FPLC system (GE Healthcare, Chicago, IL, USA) to separate the major lipoprotein classes in plasma. Fifty fractions were collected at 0.5 mL each into individual 2 mL tubes and then analyzed for TG and CHOL contents in VLDL, LDL, and HDL by colorimetric enzymatic reaction, as described above. Fraction concentrations for TG and CHOL were plotted, and the area under the curve for each lipoprotein peak representing VLDL, LDL, and HDL was calculated.

### 2.7. Western Blot

APOA1, APOA4, APOB48, and APOE were quantitated by Western blotting. Briefly, 2 μL of plasma was loaded onto 4–15% polyacrylamide gradient gels and transferred to polyvinylidene difluoride membranes. Following the transfer, membranes were blocked with 5% nonfat milk in 0.1% Tween 20 in Tris-buffered saline (TBS) for 1 h. Membranes were incubated with antibodies raised against APOA1, APOA4, APOB48, or APOE (each 1:5000) overnight at 4 °C. These antibodies have been used in our previous reports [13,14]. After rinsing with TBS with 0.1% Tween 20, membranes were incubated with peroxidase-conjugated anti-goat secondary antibody at 1:10,000 for 30 min and developed with Immobilon Western Chemiluminescent horseradish peroxidase substrate (Millipore, Billerica, MA, USA). The images from reacted membranes were acquired, and the band density was analyzed using ChemiDoc Imaging Systems (Bio-Rad Laboratories, Inc., Hercules, CA, USA). Protein quantifications were performed by densitometric analysis of films using Image Lab Software 6.0.1 (Bio-Rad Laboratories, Inc., Hercules, CA, USA). Fold changes in apolipoprotein density, including APOA1, APOA4, APOB48, and APOE, were quantified by dividing the apolipoprotein level of CV male mice.

### 2.8. Fecal DNA Extraction and 16S rRNA Gene Sequencing

16S rRNA Microseq was performed at the Michigan Microbiome Core, University of Michigan. DNA extraction from fecal pellets was performed using the Eppendorf EpMotion 5075 liquid handling system and following the Qiagen MagAttract PowerMicrobiome kit (Catalog number: 27500-4-EP) protocol. After extraction, the samples were quantified using the Quant-iT PicoGreen dsDNA Assay kit (Catalog number: P7589).

The V4 region of the 16S rRNA gene was amplified from each sample using the Dual indexing sequencing strategy by Dr. P.D. Schloss [15]. Sequencing was performed on the Illumina MiSeq platform, using a MiSeq Reagent Kit V2 500 cycles (Illumina, San Diego, CA, USA), according to the manufacturer’s instructions, with modifications found in the Schloss standard operating procedure (SOP). Accuprime High Fidelity Taq (Life Technologies, catalog number: 12346094) was used, and PCR was performed using the following parameters: 30 cycles, initial denaturation 95 °C for 2 min → denaturation 95 °C for 20 s → annealing 55 °C for 15 s → extension 72 °C for 5 min → final extension 72 °C for 10 min.

Sequencing libraries were prepared according to Illumina’s protocol for Preparing Libraries for Sequencing on the MiSeq (part number: 15,039,740 Rev. D) for 2 nM or 4 nM libraries. PhiX and genomes were added in 16S amplicon sequencing to create diversity. Sequencing reagents were prepared according to the Schloss SOP, custom read 1, read 2, and index primers were added to the reagent cartridge. FASTQ files were generated for paired-end reads.

Libraries were normalized using SequalPrep Normalization Plate Kit (Life Technologies, catalog number: A10510-01) following the manufacturer’s protocol for sequential elution. The concentration of the pooled samples was determined using the Kapa Biosystems Library Quantification kit for Illumina platforms (Kapa Biosystems KK4824). The sizes of the amplicons in the library were determined using the Agilent Bioanalyzer High Sensitivity DNA analysis kit (Catalog number: 5067-4626). The final library consisted of equal molar amounts from each plate, normalized to the pooled plate at the lowest concentration.

### 2.9. Sequence Curation

Amplicon sequence variants (ASVs) were obtained from demultiplexed fastq files using the DADA2 package (version 1.22) and algorithm [16] as implemented in QIIME2 (version qiime2-2022.8) [17]. Forward reads were truncated at 240 nucleotides, and reverse reads were truncated at 160 nucleotides. All other parameters were set to the default values. Sequencing depths ranged from 3301 to 18,011 reads per sample after error correction and chimera removal. The naive Bayesian classifier as implemented in QIIME2 was used to classify error-corrected ASVs against the SILVA 13.8 reference database trimmed to the V4 region. ASVs were inserted into the SILVA 13.8 phylogenic tree using fragment insertion via the SATé-enabled phylogenetic placement (SEPP) technique [18]. The ASV table, taxonomy, representative sequences, and phylogenetic tree were stored as a phyloseq object [19] to facilitate statistical analyses.

### 2.10. Statistical Analysis

Body weight and lipid profile data analyses. Statistical analyses were performed using GraphPad Prism V8. Data were expressed as mean values ± standard error of the mean (SEM). For comparing data with two independent variables, two-way (or repeated measures when appropriate) analysis of variance (ANOVA) with the Tukey test was used. *p*-values less than 0.05 were considered statistically significant. 

16S rDNA sequence analysis. Observed ASVs and Shannon diversity were calculated using phyloseq after subsampling to the lowest observed read depth (3301 reads). Phylogenetic diversity was calculated using the pd function in the picante package (version 1.8.2) [20]. Linear mixed effects regression (LMER) was used to test for differences in alpha-diversity from baseline (Week 0) to week 4 (Week 4), for male and female mice separately, using the LMER function in the lme4 package (version 1.1.27) [21]. Models included a random subject-specific intercept and fixed effect term for time. Standard errors and *p*-values were obtained using the Satterthwaite approximation to the model degrees of freedom as implemented in the lmerTest package (version 3.1.3) [22]. Differences in the change in alpha-diversity over time between male and female mice were obtained from the cross-product interaction term for sex and time. Ordinations of the first two principal component analysis (PCA) axes based on the Aitchison distance (i.e., centered log-ratio transformed abundance) were used to assess differences in bacterial community composition according to murine sex and time point. Testing was performed using permutational multivariate analysis of variance (PERMANOVA) as implemented in the adonis2 function in the vegan package (version 2.5.7) [23]. Models were fit as described above for the LMER, but with subject ID modeled as a fixed effect. Differences in the relative abundance of ASVs and genus-level phylotypes were tested using the linear models for differential abundance analysis of microbiome compositional data (LinDA) workflow as implemented in the LinDA package (version 0.1.0) [24]. All analyses were conducted using R version 4.1.1 [25].

## 3. Results

### 3.1. Reduced Weight Gain in Female Mice

Changes in body weight have been associated with alternations in gut microbial diversity in humans and rodents [5]. To avoid the impact of body weight changes on microbiota composition and lipid profile, we selected 4 weeks of HFD feeding period because our previous studies demonstrated that, within a relatively short period, the HFD did not significantly increase body weight in rodents [12,26].

**Figure 1:**
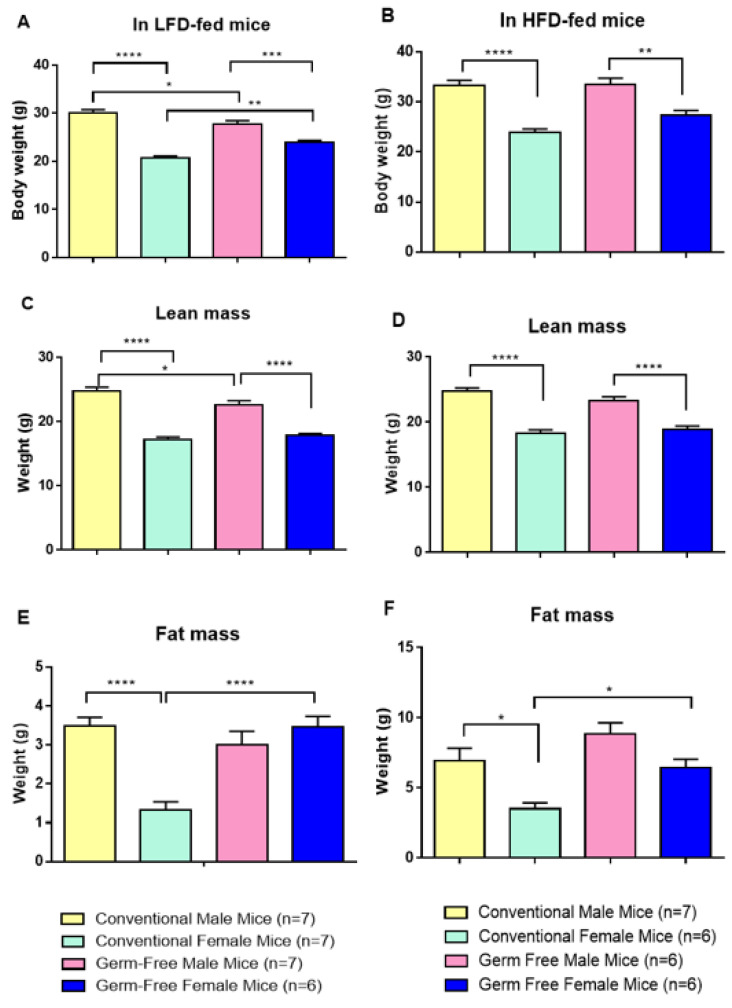
Comparison of body weight (**A**,**B**), lean mass (**C**,**D**), and fat mass (**E**,**F**) of CV and GF mice after 4-week feeding of LFD or HFD. Values are expressed as means ± SEM. * *p* < 0.05, ** *p* < 0.01, *** *p* < 0.001, **** *p* < 0.0001, compared between the groups.

Consistent with our previous observations [12,26], no significant difference in body weight was found within each group before and after individual diet consumption (Supplementary Figure S1A,B), although the body weights of HFD-fed mice displayed a tendency to increase during the 4-week feeding period. After LFD or HFD feeding, male CV and GF mice were significantly heavier than their corresponding female mice (Figure 1A,B). Lean mass in male mice was also significantly higher than that in their corresponding females (Figure 1C,D). Interestingly, female GF mice were significantly heavier than female CV mice, especially on LFD (Figure 1A), which was mainly due to the increased fat mass (Figure 1E,F), suggesting increased fat accumulation in adipose tissues of GF female mice.

### 3.2. Ameliorated Lipid Profile in Female Mice

**Figure 2:**
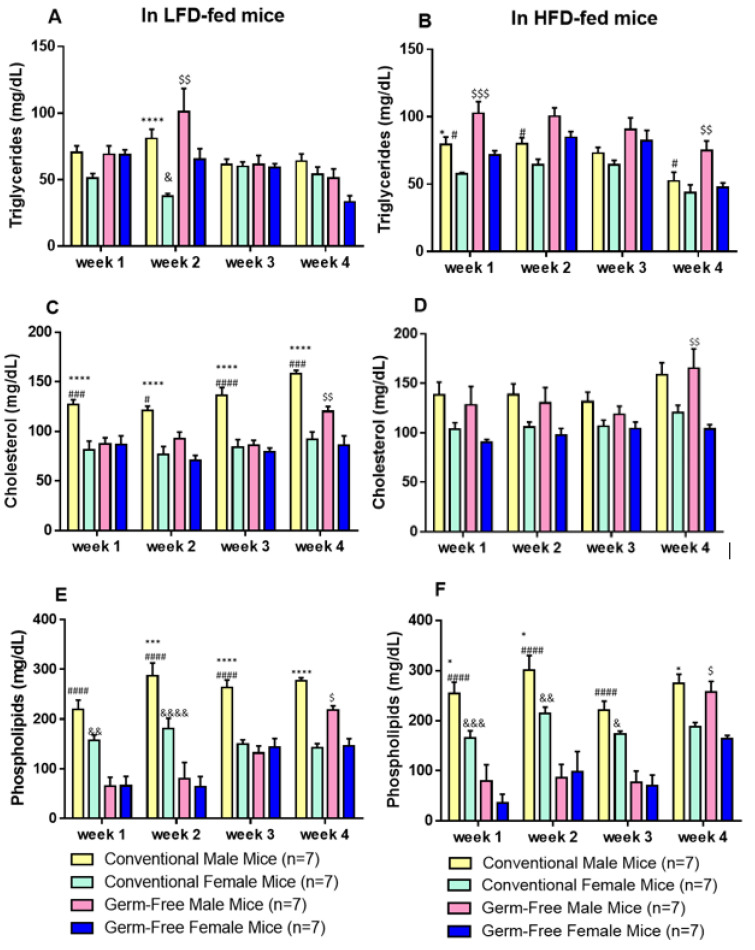
Comparison of plasma TG (**A**,**B**), CHOL (**C**,**D**), and PL (**E**,**F**) levels among CV and GF male and female mice after 4 weeks of LFD or HFD feeding. Values are expressed as means ± SEM. * *p* < 0.05, *** *p* < 0.001, and **** *p* < 0.0001, CV male mice vs. CV female mice; ^$^ *p* < 0.05, ^$$^ *p* < 0.01, and ^$$$^ *p* < 0.001, GF male mice vs. GF female mice; ^#^ *p* < 0.05, ^###^ *p* < 0.001, and ^####^ *p* < 0.0001, CV male mice vs. GF male mice; ^&^ *p* < 0.05, ^&&^ *p* < 0.01, ^&&&^ *p* < 0.001, and ^&&&&^ *p* < 0.0001, CV female mice vs. GF female mice.

Overall, TG levels were comparable between male and female mice fed LFD or HFD in the first three weeks, although there were some variations at certain time points, e.g., on Week 2 in LFD groups and Week 1 in HFD groups (Figure 2A,B). Interestingly, after 4-week HFD feeding, TG levels in GF male mice were significantly higher than those in GF female mice and in CV male mice (Figure 2B).

During 4-week LFD feeding, CHOL levels in CV male mice were significantly higher than those in the other three groups (Figure 2C). By Week 4, the GF male mice displayed significantly increased levels of CHOL than their corresponding female mice (Figure 2C). When fed an HFD, all male mice displayed a higher level of CHOL than their corresponding female mice, and by Week 4, the CHOL levels in GF male mice were significantly higher than those in GF female mice (Figure 2D).

As shown in Figure 2E,F, after LFD or HFD feeding, both male and female CV mice had significantly higher phospholipid (PL) levels in the first two weeks. By Week 4 in both LFD and HFD groups, the PL levels in GF male mice increased, and the PL levels in both male CV and GF mice were significantly higher than those in their corresponding females (Figure 2E,F).

Because Figure 1 shows lower fat mass in female mice than male mice, we also examined if female mice had greater lipolysis assessed by NEFA in plasma than male mice. No significant difference was found among four groups of mice on LFD or HFD, although there was a variation on Week 3 in LFD groups, where the CV female mice had a higher NEFA level than CV male mice (Supplementary Figure S2A,B).

### 3.3. Lipid Absorption

To investigate whether reduced fat mass in female mice resulted from altered lipid absorption, lipid absorption in female and male mice fed LFD or HFD was determined using sucrose polybehenate, as we described previously [10]. No significant difference in intestinal lipid absorption was found between CV and GF mice, regardless of diet and gender (Supplementary Figure S3A,B).

### 3.4. Lipoprotein Distribution in Plasma

**Figure 3:**
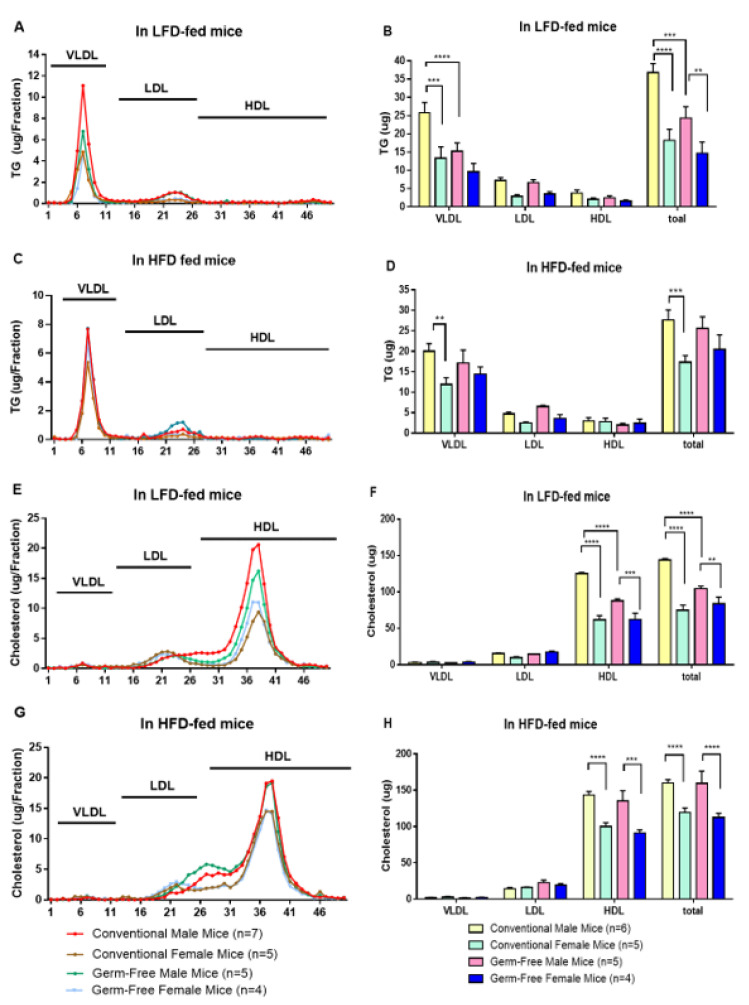
Comparison of TG (**A**–**D**) and CHOL (**E**–**H**) levels in VLDL, LDL, HDL, and total fractions among all groups after 4-week feeding with LFD or HFD. Values are expressed as means ± SEM. ** *p* < 0.01, *** *p* < 0.001, **** *p* < 0.0001 compared between the groups.

Since female mice exhibited lower plasma lipids than male mice, as shown in Figure 2, we investigated lipid-containing lipoprotein profiles in male and female GF and CV mice with LFD or HFD. In LFD groups, TG levels in both VLDL fractions and total TG levels in all fractions of CV male mice were significantly higher than those in CV female mice, as well as in GF male mice (Figure 3A,B). After 4-week HFD feeding, the differences in TG levels in both VLDL fraction and total fractions of GF male mice were increased and reached TG levels comparable to those in CV male mice (Figure 3C,D).

Unlike TG in VLDL fractions, CHOL levels were changed mainly in HDL fractions. In the LFD groups, the male CV and GF mice had significantly higher CHOL levels in HDL fractions and total CHOL levels than their corresponding female mice (Figure 3C,D). Additionally, the CV male mice had significantly higher CHOL levels in HDL fractions and total CHOL levels than GF male mice (Figure 3E,F). After 4-week HFD feeding, the levels of CHOL in HDL fractions in male GF mice were increased and were comparable to those in CV mice (Figure 3G,H).

### 3.5. Apolipoprotein Levels

**Figure 4:**
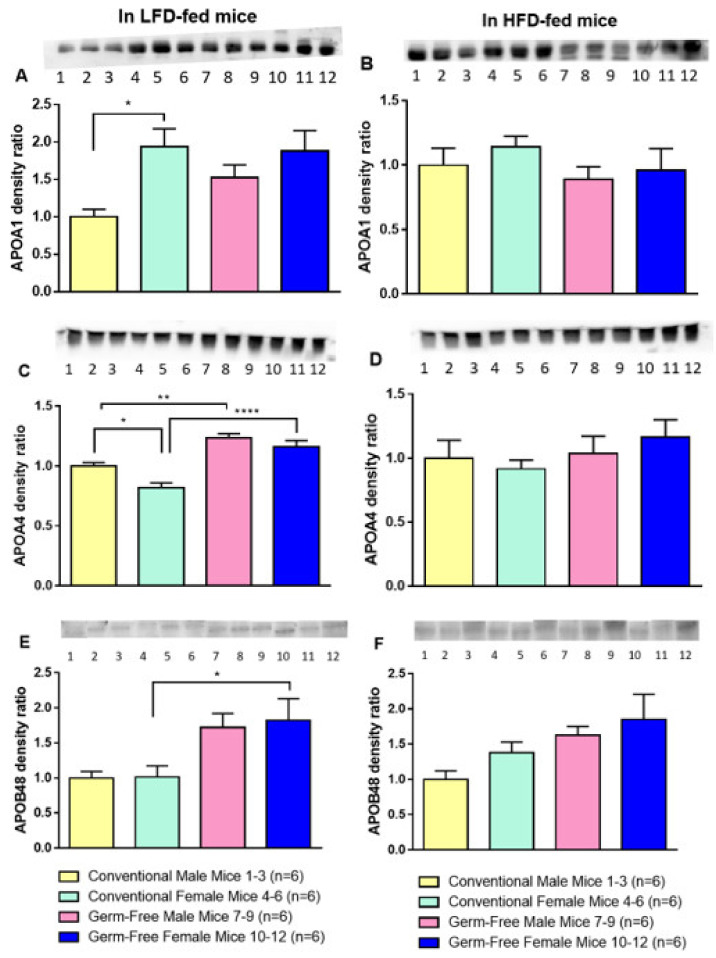
Comparison of APOA1 (**A**–**B**), APOA4 (**C**–**D**) and APOB48 (**E**–**F**) levels in plasma among all groups after 4-week feeding with LFD or HFD. Values are expressed as means ± SEM. * *p* < 0.05, ** *p* < 0.01, **** *p* < 0.0001 compared between the groups.

In the LFD group, the CV female mice had significantly higher APOA1 levels than the CV male mice. However, the difference disappeared after 4-week HFD feeding (Figure 4A,B). Additionally, no significant difference in APOA1 levels was found between male and female GF mice on LFD or HFD feeding (Figure 4A,B).

Interestingly, when on LFD, male and female GF mice had significantly higher APOA4 levels than male and female CV mice, respectively, although the APOA4 levels in male CV mice were considerably higher than those in female CV mice (Figure 4C). However, no significant differences in APOA4 levels were observed among these groups after HFD feeding (Figure 4D).

As for the levels of APOB48, the male and female GF mice fed the LFD had relatively higher levels than male and female CV mice, respectively, but the difference reached statistical significance only in female GF mice compared to CV female mice. However, all differences disappeared after HFD feeding for 4 weeks (Figure 4E,F).

We also measured plasma APOAE levels; no significant difference was found among four groups under either LFD or HFD feeding (Supplementary Figure S4A,B).

### 3.6. The Microbial Response to HFD was Dependent on Sex

To assess the impact of sex differences on the alpha-diversity of the gut microbiota, 16S rDNA from fecal samples of CV male and female mice were analyzed. Amplicon sequencing of the 16S rRNA gene was conducted to assess the changes in the murine microbiota from Week 0 to Week 4 in response to the LFD and HFD. A total of n = 24 samples were sequenced to a mean ± SD depth of 7374 ± 3320 reads per sample (range 3301; 18,011). Denoising of the raw sequence data produced 323 unique ASVs.

**Figure 5:**
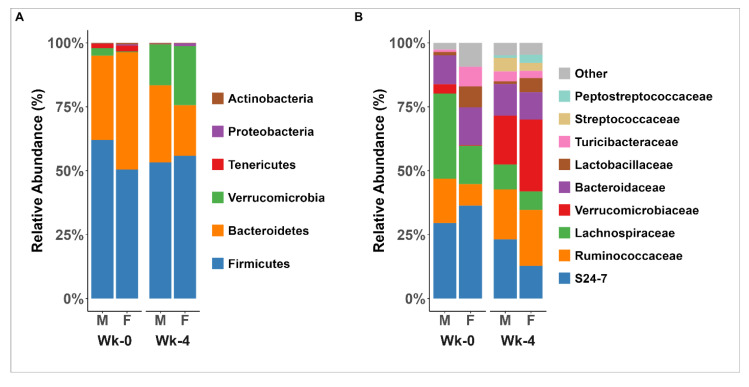
Gut microbiota was associated with sex and HFD at multiple taxa levels. Microbiota community structure at the phylum (**A**) and family levels (**B**), separated by sex and HFD feeding time (weeks). Data are shown as a relative proportion of the taxa identified. Taxa with a prevalence of >10% or with a maximum proportion of >0.2% were included. n = 6 mice per group.

Gut microbial community composition was further analyzed on read count data aggregated to phyla and families to assess differences in the relative abundances of reads mapped to these taxonomic levels (Figure 5). Analysis at the phylum level indicated that the microbiota was dominated by three phyla (>95%): *Firmicutes*, *Bacteroidetes*, and *Verrucomicrobia*. After HFD, the relative ASV abundance of phylum *Bacteroidetes* decreased, and *Verrucomicrobia* increased, especially in female mice (Figure 5A). The five most abundant families were *S24-7*, *Ruminococcaceae*, *Lachnospiraceae*, *Verrucomicrobiaceae*, and *Bacteroidaceae*, within phyla *Bacteroidetes* and *Firmicutes* (Figure 5B). HFD-induced shifts in relative abundances were evident on the level of several families. The relative ASV abundance of *S24-7* and *Lachnospiraceae* decreased and *Ruminococcaceae* and *Verrucomicrobiaceae* increased in both male and female mice (Figure 5B).

**Figure 6:**
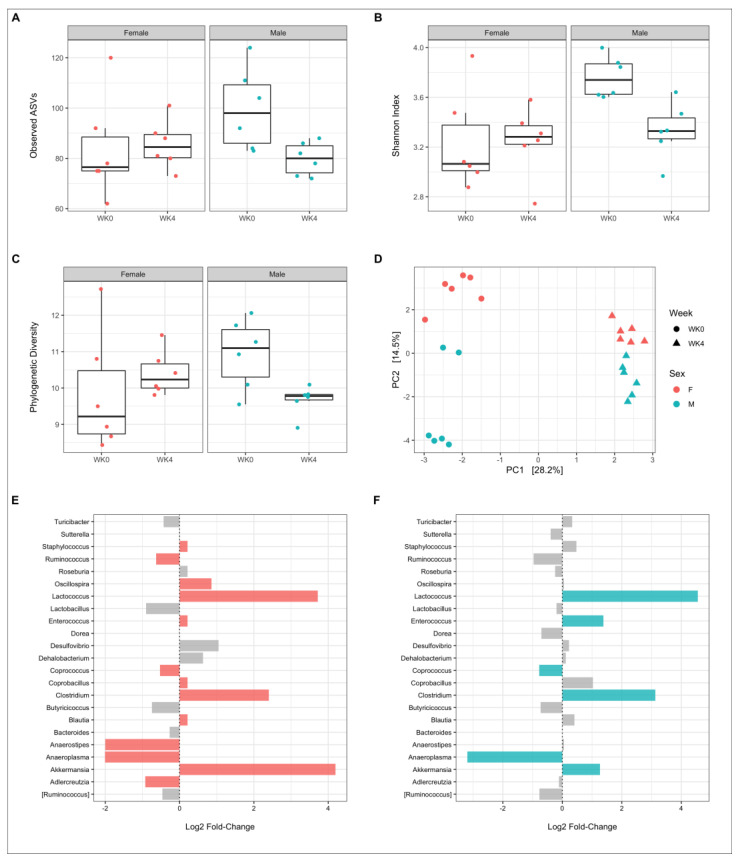
Changes in murine fecal microbiota in response to HFD intervention. (**A**) Observed amplicon sequence variants (ASVs) according to sex and study time point. (**B**) Shannon diversity according to sex and study time point. (**C**) Phylogenetic diversity according to sex and study time point. (**D**) Principal component analysis ordination performed on the distances of Aitchison (centered log-ratio transform). (**E**) Log2 fold-change for genus-level phylotypes at Week 4 compared to Week 0 in female mice. (**F**) Log2 fold-change for genus-level phylotypes at Week 4 compared to Week 0 in male mice.

Measures of alpha-diversity, including the number of observed amplicon sequence variants (ASVs) (100 ± 16 vs. 80 ± 7, *p* = 0.02), Shannon diversity (3.8 ± 0.2 vs. 3.3 ± 0.2, *p* = 0.004), and phylogenetic diversity (10.9 ± 1.0 vs. 9.7 ± 0.4, *p* = 0.03) were decreased in male mice at Week 4 when compared to Week 0 (Figure 6A–C). No statistically significant differences were observed for the change in alpha-diversity from Week 0 to Week 4 in female mice (Supplementary Table S1). The difference in the change in alpha-diversity for female versus male mice at Week 4 versus Week 0 was an increase in observed ASVs of 19.8 ± 8.2 (interaction *p* = 0.03), an increase in Shannon diversity of 0.4 ± 0.1 (interaction *p* = 0.01), and an increase in phylogenetic diversity of 1.3 ± 0.6 (interaction *p* = 0.04), which were due to corresponding decreases in males (Supplementary Table S1).

Beta-diversity ordinations performed on the Aitchison distance demonstrated clustering of samples according to sex and timepoint, highlighting differences in the overall bacterial community composition (Figure 6D). The proportion of variance explained in the Aitchison distance according to time was R^2^ = 0.37 (*p* = 0.001) and R^2^ = 0.40 (*p* = 0.001) for female and male mice, respectively, in sex-specific models. The proportion of variance attributable to the interaction for sex and time was R^2^ = 0.06 (*p* = 0.008), highlighting differences in the shift in the community composition over time for samples collected from female versus male mice.

LinDA was used to test for differences in the relative abundance of ASVs, and ASVs aggerated to genera, after a centered log-ratio transformation to account for the compositional nature of the data. The relative abundance of reads mapping *Akkermansia*, *Lactococcus*, *Clostridium*, *Oscillospira*, *Staphylococcus*, *Enterococcus*, *Coprobacillus*, and *Blautia* were enriched, and *Anaerostipes*, *Anaeroplasma*, *Adlercreutzia*, *Coprococcus*, and *Ruminococcus* depleted, in female mice at Week 4 when compared to Week 0 in response to the HFD (FDR *p* < 0.05, Figure 6E, Supplementary Table S2). A similar pattern was observed for models fit to each ASV (Supplementary Table S3). In samples from male mice, the relative abundance of reads mapping to *Lactococcus*, *Clostridium*, *Enterococcus*, and *Akkermansia* was enriched, and *Anaeroplasma* and *Coprococcus* depleted, at Week 4 in response to the HFD (Figure 6F, Supplementary Table S4). Numerous ASVs were depleted at Week 4 compared to Week 0 in male mice, consistent with the loss in alpha-diversity in response to the HFD (Supplementary Table S5). The change in the relative abundance of reads mapping to *Akkermansia* at Week 4 compared to Week 0 was more significant for females than males in models allowing for the interaction of time and sex (FDR *p* < 0.05), and the change in the relative abundance of *Anaerostipes*, *Adlercreutzia*, *Lactococcus*, and *Enterococcus* was more significant in males than females (Supplementary Table S6). Differences in the change in ASVs over time for females versus males are provided in Supplementary Table S7.

### 3.7. Select Bacterial Species Correlated to Metabolic and Lipid Parameters

**Figure 7:**
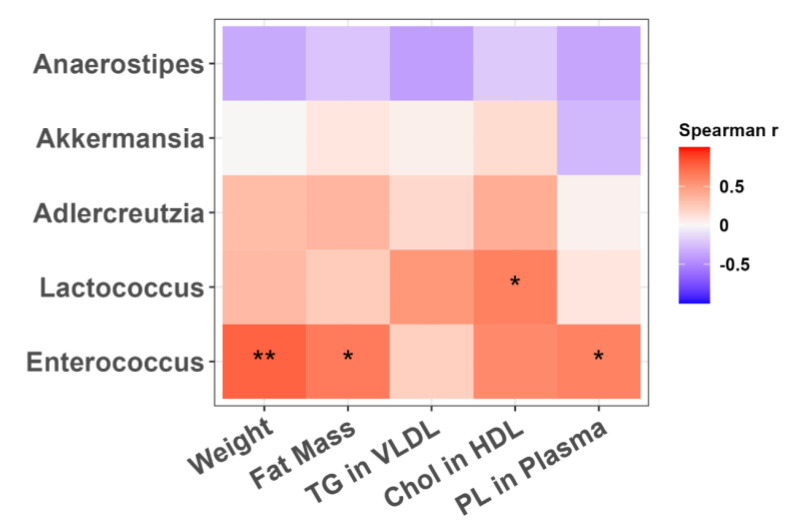
The relationship between microbiota composition and lipid parameters. * *p* < 0.05 and ** *p* < 0.01.

To comprehensively analyze the correlation between metabolic/lipid parameters and the composition of intestinal microflora, Spearman’s correlation analysis was performed. As shown in Figure 7, *Enterococcus* and *Lactococcus* were positively correlated with weight gain, fat mass, and TG in VLDL, CHOL in HDL, and PL in plasma, while *Anaerostipes* and *Akkermansia* were negatively correlated with weight gain, fat mass, and TG in VLDL, CHOL in HDL, and PL in plasma. Interestingly, the negatively related strain, *Akkermansia*, was significantly enriched in the intestine of female mice fed HFD, suggesting that *Akkermansia* might be an essential factor for the beneficial effect on dyslipidemia in females.

## 4. Discussion

Sex differences in energy and lipid metabolisms are well recognized and are associated with various common disorders, such as obesity, diabetes, and cardiovascular disease, with females generally exhibiting more beneficial metabolic profiles [27]. Now it is clear that the modern environment, e.g., the high-fat diet (HFD), substantially alters the gut microbial communities. These changes could contribute to the increased risk of metabolism-related disorders. However, it remains unclear how gender affects the interaction of host microbiota and HFD, and whether it is connected to disease susceptibility. The present studies aimed to identify sex differences in lipid absorption, blood lipid profiles, and apolipoprotein levels between CV and GF mice and to determine the modulation of microbiota composition in CV mice fed an HFD.

We and others have shown that estrogen protects female mice from HFD-induced obesity [28,29]. Estrogens exert protective effects by acting in the brain [30,31], liver [29], adipose tissue, and muscle [32] to regulate energy production and utilization. Consistent with a previous report [33], both CV and GF male mice had heavier body weight and lean mass than their corresponding female mice, regardless of whether they were on LFD or HFD. Interestingly, for the first time, we found that GF female mice were heavier than CV female mice (A,B), mainly due to significantly more fat mass (Figure 1E,F). These observations indicate that microbiota may uniquely affect fat mass composition in female mice compared with male mice.

After HFD feeding, the male CV and GF mice both exhibited higher levels of TG, CHOL, and PL in the plasma compared with their corresponding female mice (Figure 2), suggesting that male mice are susceptible to HFD, and the HFD may exert a specific effect on lipid profile regardless of microbiota. VLDL is the major TG-rich lipoprotein and is synthesized in the liver. In this study, the TG levels in the VLDL of CV male mice were found to be significantly higher than those in female mice when fed with HFD, indicating more VLDL particles or larger sizes of VLDL particles in the plasma of the male mice. HDL particles facilitate the removal of cholesterol from the plasma back to the liver. Our studies revealed higher CHOL levels in HDL of CV male mice compared with GF male mice fed with LFD, but not with HFD. This was consistent with the previous report, which showed that greater CHOL was presented in HDL of CV mice than in that of GF mice due to enhanced CHOL synthesis in the liver [34]. After HFD feeding, the difference disappeared, and overall, male mice had greater CHOL in HDL than their corresponding female mice.

APOA1 is secreted with chylomicrons, and it is quickly transferred to HDL in the circulating system [35]. As a major protein component of HDL particles in plasma, APOA1 enables the efflux of CHOL from peripheral tissues and assists CHOL delivery to LDL particles or the liver for biliary excretion [36]. APOA1 is a common biomarker for the prediction of cardiovascular disease [37]. Our previous study demonstrated that estrogen treatment significantly increased APOA1 levels in the lymph of ovariectomized animals [13]. In the current study, we found that plasma APOA1 levels in female mice were higher than those in male mice when fed LFD. The findings suggest that estrogen may increase CHOL content in HDL when mice are fed LFD.

APOA4 is a protein synthesized in the gut and secreted in association with nascent chylomicrons into intestinal lymph. The expression of APOA4 is highly responsive to intestinal lipid absorption and is required for chylomicron secretion [38]. In the present studies, we found that when fed LFD, the male and female GF mice had higher expression of APOA4 than CV mice, suggesting that gut microbiota may suppress intestinal *ApoA4* gene expression under normal metabolic status. In contrast, those differences in plasma APOA4 between GF and CV were diminished when mice were fed the HFD. No difference in plasma APOA4 level was found among the four groups, suggesting HFD may alter APOA4 levels due to modifications of microbial community composition.

APOB48, a structural protein of chylomicrons, plays a critical role in the formation and secretion of chylomicron particles [39]. It has been reported that one molecule of APOB48 is associated with one chylomicron particle [40]. The mass of lymphatic APOB48 secretion is used as a surrogate measure for the relative number of chylomicron particles [41]. In LFD-fed mice, plasma APOB48 levels in GF female mice were higher than those in CV female mice, indicating increased numbers of chylomicrons were secreted by the GF female mice. The findings suggest that the increased number of chylomicrons observed in the GF female mice may be associated with increased fat mass in the GF female mice compared to CV female mice.

HFD is a well-known risk factor for gut microbiota dysbiosis, which is characterized by an outgrowth of opportunistic pathogens with reduced beneficial bacteria [42]. *Bacteroidetes* and *Firmicutes* are the two major bacterial phyla that account for about 90% of gut microbiota, as shown in a previous report and in Figure 5 of the current study [43]. The relative proportions of the phyla *Firmicutes* to *Bacteroidetes* (F/B ratio) have been widely accepted to have an important influence in maintaining normal intestinal homeostasis [44]. Multiple studies have shown that HFD increases levels of *Firmicutes* and decreases *Bacteroidetes* [42]. According to the beta-diversity analysis in the present study (Figure 6D), sex significantly impacted gut microbial community composition and altered the relative abundance of bacteria. Specifically, the CV female mice had fewer *Firmicutes* and considerably more *Bacteroidetes* than the corresponding male mice before HFD feeding, indicating that the sex of the host impacts the gut microbiota (Figure 5A).

The differences in lipid profiles between the male and female mice were reflected in their changes in gut microbiota. As revealed by three alpha-diversity metrics, microbiota in male mice exerts a different response to HFD than that in female mice. The microbiota diversity was reduced mainly in male mice after 4-week HFD consumption (Supplementary Table S1), indicating that HFD may shape the bacteria composition, and the microbiota in male mice was susceptible to HFD, which may lead to dyslipidemia. This was consistent with previous reports that link increased weight gain with reduced gut microbiota diversity in humans and male mice [45]. There also exists sexual dimorphism in the specific microbial community composition variation after HFD. We found that the females had dramatically increased *Akkermansia* and considerably decreased *Anaerostipes*, *Adlercreutzia*, *Lactococcus*, and *Enterococcus* compared to male mice (Figure 6E,F, as well as Supplementary Table S6).

*Akkermansia*, a mucin-degrading bacteria, is usually found in 1–4% abundance in the human gut [46]. A previous study found that *Akkermansia* alleviated endoplasmic reticulum stress and reduced the expression of genes involved in fatty acid synthesis and transport in both the liver and muscle, leading to improved lipid accumulation and metabolic disorders [47]. Additionally, *Akkermansia* increased thermogenesis by stimulating uncoupling protein 1 gene expression in brown adipose tissue, improved glucose homeostasis, and ameliorated metabolic disease by stimulating glucagon-like peptide-1 (GLP-1) secretion in C57BL/6 mice after HFD consumption [48]. More recently, it was reported that *Akkermansia* was negatively correlated with total cholesterol and low-density lipoprotein cholesterol levels in mice fed HFD [49]. Thus, the findings suggest that the considerable enrichment of *Akkermansia* observed in female mice could be a key factor for protecting from dyslipidemia at the microbiota level in female mice fed HFD.

Unlike the enrichment of bacteria described above, certain bacteria decreased in female mice after HFD feeding, such as *Adlercreutzia*, *Anaerostipes*, and *Enterococcus*. *Adlercreutzia* was found to be enhanced in HFD rats [29] and positively associated with total fat gain [50], suggesting that after HFD, more bacteria related to obesity were altered in male mice than female mice, possibly attributing to susceptibility to HFD-induced obesity. *Anaerostipes* is a butyrate-producing genus that is associated with the gut microbiota of healthy hosts [51]. *Anaerostipes* was found to be positively correlated with total cholesterol [52], which is consistent with what we found in HFD-fed female mice. Those mice had lower total CHOL levels with less abundant *Anaerostipes*. Another important finding in this study was that male mice had an increased abundance of *Enterococcus*, which was positively associated with inflammation [53]. Contrarily, the abundance of *Enterococcus* was markedly reduced in females (Figure 6E,F). Inflammation induced by HFD is a factor contributing to metabolic disorders, and compelling evidence suggests that inflammation is a critical mechanism by which obesity promotes the progression of inflammatory diseases, such as dyslipidemia, nonalcoholic fatty liver disease, and type 2 diabetes mellitus [54]. In fact, significantly increased *Enterococcus* was found in obese rats compared to lean rats, and the concentration of *Enterococcus* was positively correlated with fat pad mass, body mass, mass gain, and food intake [55].

Gender-based effects under gut microbiota conditions are always neglected. Actually, previous studies have revealed that gender has a close relation with beneficial commensal enrichment after a certain diet [56,57]. In addition, certain metabolites digested by microbiota, such as polyphenol metabolites, are also altered in different genders [58,59]. In addition, different dietary contents also alter microbiota composition in sex-specific patterns [60,61,62]. So, in the future, more attention should be paid to the interplay between sex differences in microbiota and related metabolism.

## 5. Conclusions

In conclusion, our findings demonstrated significant sex differences in lipid metabolism and the microbiota composition at baseline (during LFD), along with sex-dependent responses to HFD. Our findings highlighted the importance of considering sex in designing feeding studies. However, the mechanisms underlying these effects are still obscure. Sex hormones, e.g., estrogen and testosterone, are the factors that most likely mediate gender-dependent interactions. Further studies, such as gonadectomy or hormone replacement in mice, need to be conducted to investigate sex hormones’ role in regulating microbial community composition and lipid metabolism. Additionally, since bile acids have been shown to affect gut microbiota [63], future studies should also analyze and compare bile acid composition between male and female mice after HFD feeding to determine if it is a possible mechanism for sex differences. Given that many metabolic diseases are characterized by disturbances in the gut microbiome, investigating how sex-dependent lipid regulation is associated with disruption in microbial homeostasis will provide a foundation for future functional studies on the role of sex hormones and the gut microbiome in metabolic disorders in women.

## Data Availability

Not available.

## Supplementary Materials

The following supporting information can be downloaded at: https://www.mdpi.com/article/10.3390/nu15092175/s1, Figure S1: Body weight changes of the mice during the four-week feeding period with LFD. Figure S2: Comparison of plasma NEFA levels among CV and GF mice after 4-week feeding with LFD or HFD. Figure S3: Comparison of lipid absorption among CV and GF mice after 4-week feeding with LFD or HFD. Figure S4: Comparison of plasma APOE levels in the mice after LFD and HFD feeding for 4 weeks. Table S1: Alpha-diversity according to time and sex. Table S2: The difference in relative abundance of genus-level phylotypes in female mice at Week 4 when compared to Week 0 (before HFD feeding). Table S3: The difference in relative abundance of amplicon sequence variants (ASVs) in female mice at Week 4 when compared to Week 0 in response to the HFD. Table S4: The difference in relative abundance of genus-level phylotypes in male mice at Week 4 when compared to Week 0 in response to the HFD. Table S5: The difference in relative abundance of amplicon sequence variants (ASVs) in male mice at Week 4 when compared to Week 0 in response to the HFD. Table S6: The difference in relative abundance of genus-level phylotypes at Week 4 vs. Week 0 for female mice when compared to male mice in response to the HFD. Table S7: The difference in relative abundance of amplicon sequence variants (ASVs) at Week 4 vs. Week 0 for female mice when compared to male mice in response to the HFD.

## Abbreviations

TG	triglyceride
CHOL	cholesterol
PL	phospholipid
NEFA	non-esterified fatty acid
HFD	high-fat diet
LFD	low-fat diet
VLDL	very-low-density lipoprotein
HDL	high-density lipoprotein
lfcSE	log2 fold-change standard error
padj	FDR corrected *p*-value
df	degrees of freedom
