# Supplementary material for: Sexual Dimorphism in Lipid Metabolism and Gut Microbiota in Mice Fed a High-Fat Diet
Source: Nutrients. 2023 May 2;15(9):2175. doi: 10.3390/nu15092175 (PMC10180580; doi:10.3390/nu15092175)
Supplement: Supplementary file 1 [file nutrients-15-02175-s001.zip › nutrients-2356984-supplementary.pdf]

Supplemental figures:

Figure S1: Body weight changes of the mice during the four-week feeding period with LFD (A) and HFD (B). No significant difference was found within each group before and after the consumption of individual diets.

Figure S2: Comparison of plasma NEFA (A, B) levels among CV and GF mice after Week 4 feeding with LFD and HFD, respectively. Values are expressed as means ± SEM. $^{*}P < 0.05$, comparison of CV male mice to female mice.

Figure S3: Comparison of lipid absorption among CV and GF mice after Week 4 feeding with LFD and HFD, respectively. Values are expressed as means ± SEM.

Figure S4: Comparison of plasma APOE levels in the mice after LFD and HFD feeding for 4 weeks. Values are expressed as means ± SEM.

Supplemental Figures

Figure S1:

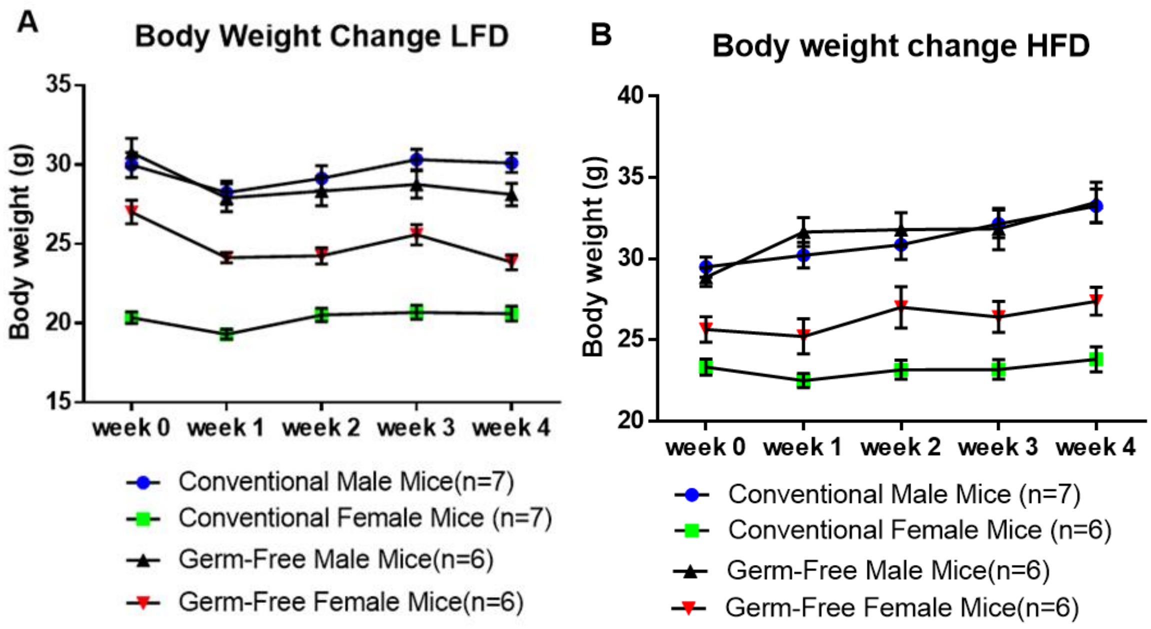

Figure S2:

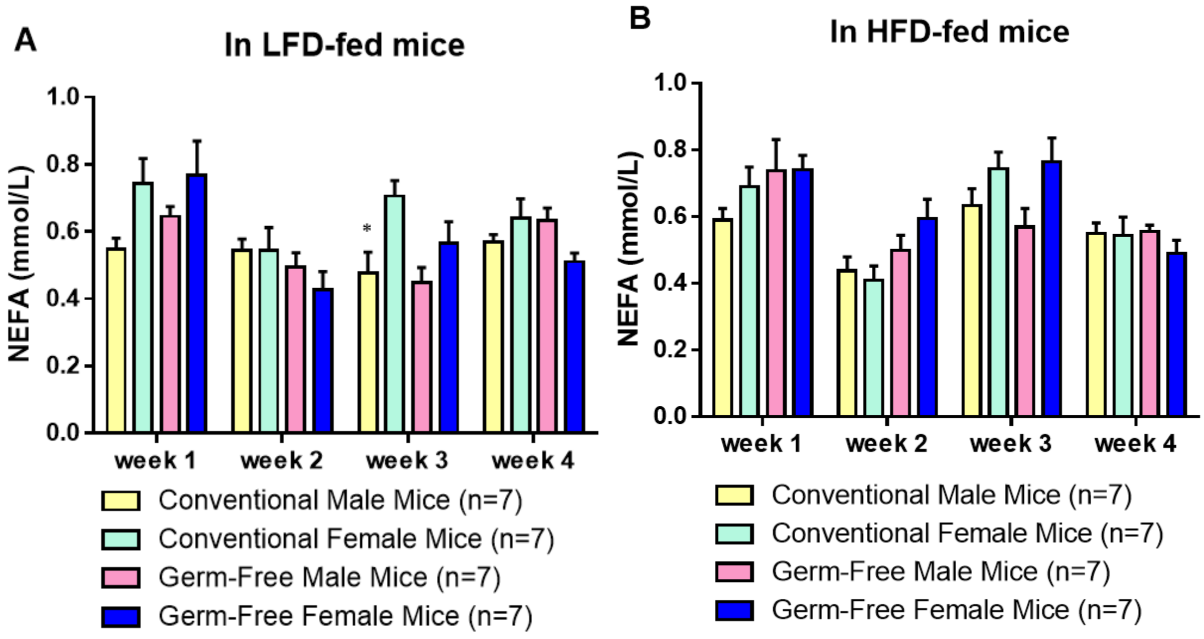
A
In LFD-fed mice
NEFA (mmol/L)
1.0
0.8
0.6
0.4
0.2
0.0
*
week 1
week 2
week 3
week 4
Conventional Male Mice (n=7)
Conventional Female Mice (n=7)
Germ-Free Male Mice (n=7)
Germ-Free Female Mice (n=7)
B
In HFD-fed mice
NEFA (mmol/L)
1.0
0.8
0.6
0.4
0.2
0.0
week 1
week 2
week 3
week 4
Conventional Male Mice (n=7)
Conventional Female Mice (n=7)
Germ-Free Male Mice (n=7)
Germ-Free Female Mice (n=7)

Supplemental Figures

Figure S3

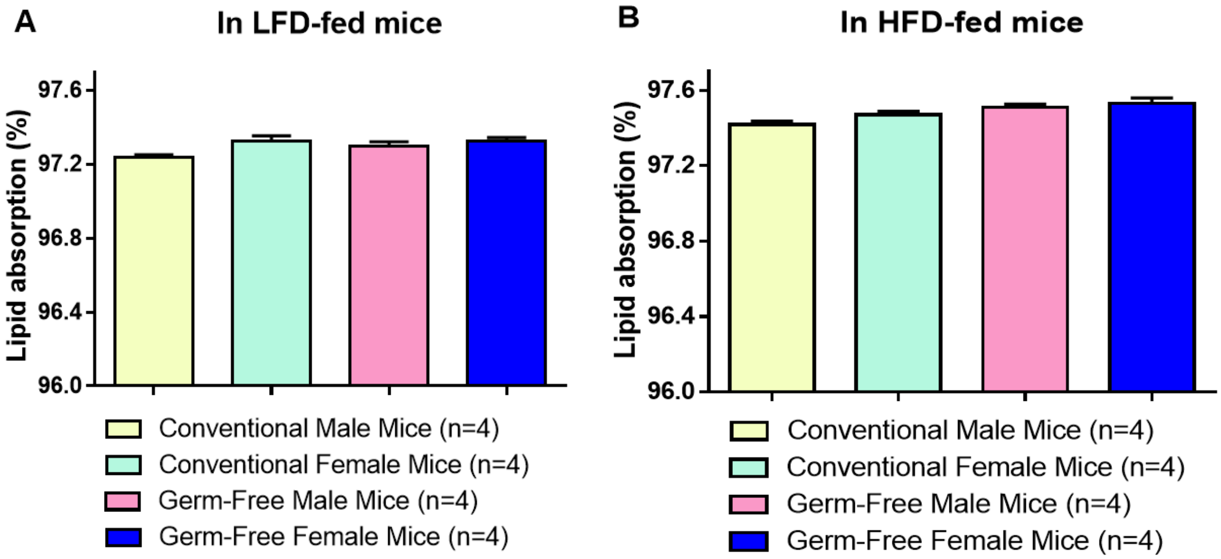

Supplemental Figures

Figure S4

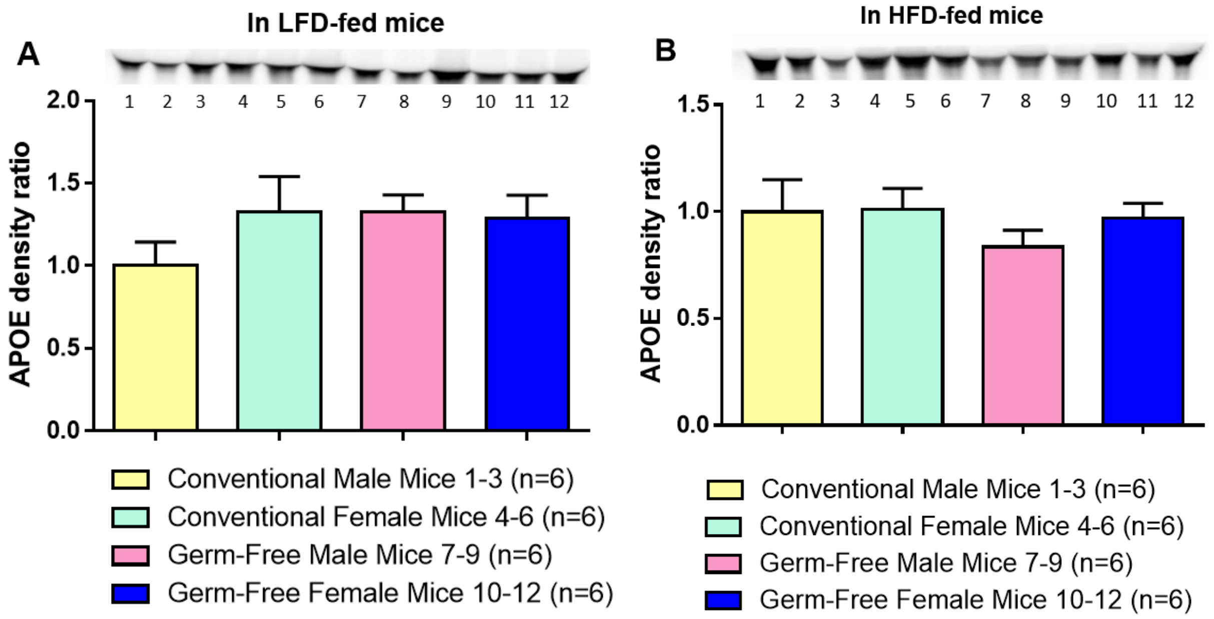

Supplemental Table S1. Alpha-diversity according to time and sex

Alpha diversity	Week-0	Week-4	P-value for wk-0/wk-4 difference	P-value for week by sex interaction*
**Observed**				
Female	83.67 (20.21)	85.50 (9.73)	0.845	0.026
Male	99.67 (16.26)	79.83 (6.65)	0.020	
**Shannon Index**				
Female	3.24 (0.40)	3.25 (0.28)	0.168	0.013
Male	3.76 (0.17)	3.33 (0.23)	0.004	
**Faith's Phylogenetic Diversity**				
Female	9.84 (1.64)	10.41 (0.61)	0.447	0.044
Male	10.94 (0.96)	9.67 (0.40)	0.029	

Notes. P-values obtained from linear mixed effects regression. Values at week 0 and week 4 reflect the mean (standard deviation).

Supplemental Table S2. The difference in the relative abundance of genus-level phylotypes in female mice at Week 4 when compared to Week 0 (before HFD feeding)

Genus	baseMean	log2FoldChange	lfcSE	stat	pvalue	padj	reject	df
Anaerostipes	2453.355972	-1.999488	0.145847	-13.709514	8.27E-08	1.90E-06	TRUE	10.0
Lactococcus	6187.902464	3.719104	0.163698	22.719274	1.04E-06	1.19E-05	TRUE	5.6
Clostridium	3603.687518	2.403466	0.326781	7.354974	2.44E-05	1.87E-04	TRUE	10.0
Akkermansia	42954.88472	4.195634	0.506415	8.284968	2.00E-04	0.00115	TRUE	5.8
Adlercreutzia	10614.10675	-0.925339	0.089366	-10.354521	5.32E-04	0.00245	TRUE	3.9
Oscillospira	238644.4629	0.858399	0.117261	7.320416	0.001620	0.0062	TRUE	4.1
Anaeroplasma	11461.37974	-2.005654	0.470646	-4.261491	0.005610	0.01843	TRUE	5.9
Coprococcus	25097.41256	-0.530003	0.157667	-3.361535	0.007220	0.02077	TRUE	10.0
Ruminococcus	43383.19318	-0.631346	0.156927	-4.023179	0.008300	0.0212	TRUE	5.5
Staphylococcus	176.088433	0.211941	0.060671	3.493286	0.015920	0.02817	TRUE	5.3
Enterococcus	176.088433	0.211941	0.060671	3.493286	0.015920	0.02817	TRUE	5.3
Coprobacillus	176.088433	0.211941	0.060671	3.493286	0.015920	0.02817	TRUE	5.3
Blautia	176.088433	0.211941	0.060671	3.493286	0.015920	0.02817	TRUE	5.3
Roseburia	275.100344	0.214572	0.066373	3.232818	0.032360	0.05316	FALSE	4.0
[Ruminococcus]	68861.3206	-0.460339	0.163726	-2.811645	0.044950	0.06892	FALSE	4.3
Lactobacillus	105094.7944	-0.902418	0.476608	-1.893417	0.124230	0.17858	FALSE	4.4
Dehalobacterium	6973.915896	0.629383	0.359457	1.750929	0.144500	0.1955	FALSE	4.7
Bacteroides	358856.4558	-0.270255	0.198642	-1.360515	0.227690	0.29093	FALSE	5.4
Desulfovibrio	309.85493	1.048745	0.846668	1.238672	0.243750	0.29507	FALSE	10.0
Butyricicoccus	698.035366	-0.743471	0.731283	-1.016668	0.333280	0.38327	FALSE	10.0
Turicibacter	65010.97271	-0.430061	0.520448	-0.826329	0.444380	0.4867	FALSE	5.3
Sutterella	8303.343243	0.008513	0.653779	0.013021	0.990030	0.99003	FALSE	6.0
Dorea	511.467255	-0.009764	0.706317	-0.013823	0.989240	0.99003	FALSE	10.0

Abbreviations: lfcSE, log2FoldChange standard error; padj, FDR corrected p-value; df, degrees of freedom.
Notes. Estimates were obtained from bias-corrected centered log2-ratio transformed regression as implemented in LinDA.
baseMean reflects the normalized average read count. Stat is the log2FoldChange / lfcSE.
Model degrees of freedom computed using the Satterthwaite approximation as implemented in lmerTest.

Supplemental Table S3. The difference in the relative abundance of amplicon sequence variants (ASV) in female mice at Week 4 compared to Week 0 in response to the HFD.

ASV	baseMean	log2FoldChange	lfcSE	stat	pvalue	padj	df	Kingdom	Phylum	Class	Order	Family	Genus	Species
ASV112	1618.631262	-3.328240472	0.161436979	-20.61634517	1.59E-09	2.26E-07	10	Bacteria	Firmicutes	Clostridia	Clostridiales			
ASV100	1746.414598	-3.442736635	0.187033512	-18.40705764	4.82E-09	3.42E-07	10	Bacteria	Firmicutes	Clostridia	Clostridiales	Ruminococcaceae	Ruminococcus	
ASV113	1924.402013	-3.588976613	0.262308167	-13.6822908	8.43E-08	3.99E-06	10	Bacteria	Firmicutes	Clostridia	Clostridiales			
ASV171	1853.054338	2.248433939	0.185183531	12.14165171	2.62E-07	9.29E-06	10	Bacteria	Firmicutes	Clostridia	Clostridiales	Lachnospiraceae	Coprococcus	
ASV134	8857.924455	4.003650139	0.369293757	10.84136966	7.54E-07	2.14E-05	10	Bacteria	Firmicutes	Clostridia	Clostridiales			
ASV95	1292.122671	1.705142298	0.168992047	10.09007421	1.46E-06	3.46E-05	10	Bacteria	Firmicutes	Clostridia	Clostridiales	Ruminococcaceae		
ASV104	4362.713571	-3.377591547	0.367306289	-9.195572327	3.41E-06	6.92E-05	10	Bacteria	Firmicutes	Clostridia	Clostridiales	Ruminococcaceae	Ruminococcus	
ASV294	2753.546545	-4.140855912	0.116893458	-35.42418861	5.62E-06	9.97E-05	3.843	Bacteria	Firmicutes	Clostridia	Clostridiales	Lachnospiraceae		
ASV61	3430.99881	3.146588093	0.200547247	15.69000894	1.29E-05	1.99E-04	5.255	Bacteria	Firmicutes	Clostridia	Clostridiales	Ruminococcaceae	Oscillospira	
ASV205	4809.354345	3.655408976	0.238812287	15.30662023	1.40E-05	1.99E-04	5.284	Bacteria	Firmicutes	Clostridia	Clostridiales	Peptostreptococcaceae		
ASV41	4926.262509	3.691116824	0.242728411	15.20677702	1.82E-05	2.15E-04	5.134	Bacteria	Firmicutes	Bacilli	Lactobacillales	Streptococcaceae	Lactococcus	
ASV262	3361.433442	-4.405583104	0.310793209	-14.17528753	1.69E-05	2.15E-04	5.433	Bacteria	Firmicutes	Clostridia	Clostridiales			
ASV163	2730.959559	2.824612008	0.254889806	11.08169862	5.26E-05	4.98E-04	5.572	Bacteria	Firmicutes	Clostridia	Clostridiales			
ASV194	2353.716148	1.871114392	0.096691411	19.35140223	4.91E-05	4.98E-04	3.918	Bacteria	Firmicutes	Clostridia	Clostridiales	Lachnospiraceae		
ASV210	1789.112318	2.151684711	0.1467915	14.65810153	5.05E-05	4.98E-04	4.579	Bacteria	Firmicutes	Clostridia	Clostridiales	Ruminococcaceae	Ruminococcus	
ASV146	1613.543957	-3.323497048	0.553813998	-6.001106977	1.32E-04	0.001102	10	Bacteria	Firmicutes	Clostridia	Clostridiales			
ASV168	1001.695015	-2.60356668	0.285667965	-9.113960966	1.31E-04	0.001102	5.699	Bacteria	Firmicutes	Clostridia	Clostridiales	Lachnospiraceae	Coprococcus	
ASV92	808.6461546	-2.983344259	0.191831165	-15.55192694	1.44E-04	0.001108	3.783	Bacteria	Firmicutes	Clostridia	Clostridiales	Ruminococcaceae	Ruminococcus	
ASV323	1233.737284	-2.919082275	0.49358639	-5.914025056	1.48E-04	0.001108	10	Bacteria	Firmicutes	Clostridia	Clostridiales	Lachnospiraceae		
ASV19	6321.369436	-1.454031212	0.108371143	-13.41714379	1.77E-04	0.001171	4.006	Bacteria	Bacteroidetes	Bacteroidia	Bacteroidales	Rikenellaceae		
ASV129	1160.571767	-2.798369687	0.322885197	-8.666763658	1.86E-04	0.001171	5.616	Bacteria	Firmicutes	Clostridia	Clostridiales			
ASV258	3078.020674	2.301549135	0.40078258	5.742637659	1.87E-04	0.001171	10	Bacteria	Firmicutes	Erysipelotrichi	Erysipelotrichales	Erysipelotrichaceae	Clostridium	cocleatum
ASV281	1338.344372	-3.041717377	0.530631307	-5.73226143	1.90E-04	0.001171	10	Bacteria	Firmicutes	Clostridia	Clostridiales	Lachnospiraceae	[Ruminococcus]	gnavus
ASV193	1178.179102	-2.849649995	0.503610892	-5.658435992	2.10E-04	0.001242	10	Bacteria	Firmicutes	Clostridia	Clostridiales	Lachnospiraceae		
ASV182	1245.016306	-2.901341651	0.371695814	-7.805688267	3.04E-04	0.001726	5.682	Bacteria	Firmicutes	Clostridia	Clostridiales	Lachnospiraceae		
ASV266	2132.544737	-2.687440891	0.23315452	-11.52643701	4.23E-04	0.002308	3.809	Bacteria	Firmicutes	Clostridia	Clostridiales	Lachnospiraceae	Aerostipes	
ASV147	8420.485958	-4.20168718	0.550315456	-7.635052101	4.43E-04	0.002318	5.373	Bacteria	Firmicutes	Clostridia	Clostridiales			
ASV296	2657.779608	2.027282258	0.396707782	5.110265914	4.57E-04	0.002318	10	Bacteria	Firmicutes	Clostridia	Clostridiales	Lachnospiraceae		
ASV309	6263.650945	2.544407984	0.522746471	4.867384334	6.54E-04	0.003203	10	Bacteria	Firmicutes	Clostridia	Clostridiales	Lachnospiraceae		
ASV21	37732.41144	4.026691593	0.621942159	6.474382758	7.44E-04	0.003523	5.787	Bacteria	Verrucomicrobia	Verrucomicrobiae	Verrucomicrobiales	Verrucomicrobiaceae	Akkermansia	muciniphila
ASV318	3353.232326	-2.482174252	0.376046127	-6.600717501	7.70E-04	0.003527	5.598	Bacteria	Firmicutes	Clostridia	Clostridiales	Lachnospiraceae		
ASV165	5295.004387	-2.801663216	0.612979038	-4.570569369	0.001025	0.00455	10	Bacteria	Firmicutes	Clostridia	Clostridiales	Lachnospiraceae	Coprococcus	
ASV27	10946.52817	-2.413218423	0.53561841	-4.505480729	0.001133	0.004877	10	Bacteria	Tenericutes	Mollicutes	Aeroplasmatales	Aeroplasmataceae	Aeroplasma	
ASV80	1177.008629	-2.207822855	0.364331515	-6.059928286	0.001588	0.006634	5.153	Bacteria	Actinobacteria	Coriobacteriia	Coriobacteriales	Coriobacteriaceae	Adlercreutzia	
ASV136	3313.238415	-2.600655905	0.610289097	-4.261350759	0.001659	0.006733	10	Bacteria	Firmicutes	Clostridia	Clostridiales			
ASV223	6075.140659	2.176575508	0.518853313	4.194972748	0.001843	0.007271	10	Bacteria	Firmicutes	Clostridia	Clostridiales	Ruminococcaceae	Oscillospira	
ASV11	9137.623951	-2.409456862	0.337600335	-7.137009684	0.001968	0.007554	4.037	Bacteria	Bacteroidetes	Bacteroidia	Bacteroidales	S24-7		
ASV138	684.0113338	-1.977937093	0.356810584	-5.543381227	0.002231	0.008336	5.262	Bacteria	Firmicutes	Clostridia	Clostridiales			
ASV46	469.4693176	-1.463167328	0.389194861	-3.759472374	0.003725	0.013564	10	Bacteria	Firmicutes	Clostridia	Clostridiales	[Mogibacteriaceae]		
ASV192	1774.939416	-2.524188511	0.49153127	-5.135356921	0.004112	0.014599	4.8	Bacteria	Firmicutes	Clostridia	Clostridiales	Lachnospiraceae		
ASV195	612.3737212	-1.863596246	0.523987537	-3.556565973	0.005211	0.017919	10	Bacteria	Firmicutes	Clostridia	Clostridiales	Lachnospiraceae		
ASV219	2058.97341	2.407213663	0.678776368	3.546401696	0.0053	0.017919	10	Bacteria	Firmicutes	Clostridia	Clostridiales	Ruminococcaceae	Oscillospira	
ASV126	1795.334062	2.149459634	0.496118029	4.332556992	0.005506	0.018183	5.713	Bacteria	Firmicutes	Clostridia	Clostridiales			
ASV97	2495.510421	2.112765644	0.279323535	7.563865471	0.005787	0.018676	2.839	Bacteria	Firmicutes	Clostridia	Clostridiales	Ruminococcaceae		
ASV175	1919.946408	-2.657065412	0.609571079	-4.358909903	0.006897	0.021763	5.124	Bacteria	Firmicutes	Clostridia	Clostridiales	Lachnospiraceae		
ASV216	16050.9536	-0.875866987	0.196653359	-4.453862334	0.00719	0.022195	4.846	Bacteria	Firmicutes	Clostridia	Clostridiales	Ruminococcaceae	Oscillospira	
ASV78	16489.23769	2.485496796	0.603026056	4.121707134	0.007646	0.022619	5.443	Bacteria	Firmicutes	Clostridia	Clostridiales	Ruminococcaceae		
ASV228	1487.350807	1.910270038	0.486387694	3.927463753	0.007585	0.022619	6.059	Bacteria	Firmicutes	Clostridia	Clostridiales	Ruminococcaceae	Oscillospira	
ASV284	8656.932758	-2.067263289	0.488849672	-4.22883231	0.008835	0.025603	4.848	Bacteria	Firmicutes	Clostridia	Clostridiales	Lachnospiraceae	[Ruminococcus]	gnavus
ASV189	2197.499129	-1.959597414	0.615810451	-3.182143808	0.009784	0.027786	10	Bacteria	Firmicutes	Clostridia	Clostridiales	Lachnospiraceae		
ASV261	699.0221969	-2.023092387	0.543413748	-3.72293192	0.011573	0.032223	5.481	Bacteria	Firmicutes	Clostridia	Clostridiales			
ASV321	708.9495803	-2.084261247	0.693289911	-3.006334313	0.0132	0.036046	10	Bacteria	Firmicutes	Clostridia	Clostridiales	Lachnospiraceae		
ASV76	2063.190528	-2.49793382	0.844084874	-2.959339632	0.014304	0.038325	10	Bacteria	Firmicutes	Clostridia	Clostridiales			
ASV70	533.5901995	-1.749685529	0.435814145	-4.014751588	0.01493	0.038547	4.133	Bacteria	Firmicutes	Clostridia	Clostridiales	Ruminococcaceae	Oscillospira	
ASV190	618.7562005	-2.02954177	0.526900893	-3.851847279	0.014822	0.038547	4.471	Bacteria	Firmicutes	Clostridia	Clostridiales	Lachnospiraceae		
ASV39	3373.695713	-1.965842242	0.528623426	-3.71879517	0.015644	0.03967	4.651	Bacteria	Firmicutes	Bacilli	Lactobacillales	Lactobacillaceae	Lactobacillus	
ASV211	658.5563661	-1.911043264	0.504931085	-3.784760575	0.016766	0.041769	4.326	Bacteria	Firmicutes	Clostridia	Clostridiales	Ruminococcaceae	Ruminococcus	
ASV292	743.2140061	-1.666686038	0.583892689	-2.854438955	0.017119	0.041913	10	Bacteria	Firmicutes	Clostridia	Clostridiales			
ASV181	587.8837049	-1.875647964	0.503744085	-3.723414366	0.017989	0.043296	4.293	Bacteria	Firmicutes	Clostridia	Clostridiales	Lachnospiraceae		
ASV173	654.7532192	-1.887023924	0.499222111	-3.779928577	0.019793	0.046844	3.96	Bacteria	Firmicutes	Clostridia	Clostridiales	Lachnospiraceae		
ASV84	4295.707975	-1.544742633	0.419533245	-3.682050595	0.023368	0.054397	3.78	Bacteria	Actinobacteria	Coriobacteriia	Coriobacteriales	Coriobacteriaceae	Adlercreutzia	
ASV127	2232.172766	1.740274962	0.583158834	2.984221212	0.027661	0.062347	5.432	Bacteria	Firmicutes	Clostridia	Clostridiales			
ASV252	996.1166779	-1.508905689	0.472862817	-3.191000934	0.027335	0.062347	4.59	Bacteria	Firmicutes	Clostridia	Clostridiales			
ASV268	1316.689955	-2.2080118	0.734857611	-3.004679775	0.029222	0.064835	5.097	Bacteria	Firmicutes	Clostridia	Clostridiales	Lachnospiraceae	[Ruminococcus]	gnavus
ASV153	1492.532945	1.895737328	0.176079932	10.76634518	0.035189	0.076875	1.244	Bacteria	Firmicutes	Clostridia	Clostridiales	Lachnospiraceae		
ASV298	7597.124174	-2.35981757	0.99419537	-2.373595412	0.039037	0.083989	10	Bacteria	Firmicutes	Clostridia	Clostridiales			
ASV18	9259.900056	-1.43908734	0.487775464	-2.950306945	0.040772	0.086412	4.093	Bacteria	Bacteroidetes	Bacteroidia	Bacteroidales	S24-7		
ASV109	1546.61387	-1.37429102	0.498574967	-2.756438071	0.042639	0.08904	4.713	Bacteria	Firmicutes	Clostridia	Clostridiales			
ASV130	2020.72692	2.368857063	0.929036197	2.549800612	0.045271	0.093166	5.736	Bacteria	Firmicutes	Clostridia	Clostridiales			
ASV279	461.6838675	-0.949271247	0.425083487	-2.233140725	0.049578	0.100572	10	Bacteria	Firmicutes	Clostridia	Clostridiales	Lachnospiraceae		
ASV143	1105.230815	-1.365745227	0.542396143	-2.517984767	0.050994	0.101987	5.254	Bacteria	Firmicutes	Clostridia	Clostridiales			
ASV128	6636.337593	1.352861467	0.613675965	2.204520862	0.052041	0.102637	10	Bacteria	Firmicutes	Clostridia	Clostridiales			
ASV40	82816.3222	-1.100496021	0.441885199	-2.49045685	0.058871	0.114025	4.644	Bacteria	Firmicutes	Bacilli	Lactobacillales	Lactobacillaceae	Lactobacillus	salivarius
ASV183	4575.301988	-1.561956108	0.734697181	-2.125986254	0.059422	0.114025	10	Bacteria	Firmicutes	Clostridia	Clostridiales	Lachnospiraceae		
ASV141	3096.738132	1.924287472	0.868713073	2.215101318	0.06762	0.128027	6.144	Bacteria	Firmicutes	Clostridia	Clostridiales			
ASV142	8071.171866	1.410641588	0.636042945	2.21784016	0.069825	0.130463	5.814	Bacteria	Firmicutes	Clostridia	Clostridiales			
ASV3	344579.9721	-0.593759613	0.27136605	-2.188039415	0.075407	0.139063	5.495	Bacteria	Bacteroidetes	Bacteroidia	Bacteroidales	Bacteroidaceae	Bacteroides	
ASV63	39699.78599	0.590592206	0.312277359	1.891242474	0.087882	0.15999	10	Bacteria	Firmicutes	Clostridia	Clostridiales	Ruminococcaceae	Oscillospira	
ASV73	903.0851932	-1.202593266	0.66675818	-1.803642312	0.101448	0.180071	10	Bacteria	Firmicutes	Clostridia	Clostridiales	Ruminococcaceae	Butyricicoccus	pullicaecorum
ASV208	561.8686368	-1.053792629	0.583558774	-1.805803763	0.101092	0.180071	10	Bacteria	Firmicutes	Clostridia	Clostridiales	Ruminococcaceae	Ruminococcus	
ASV116	954.6751609	1.249064534	0.704380446	1.773281103	0.106583	0.18685	10	Bacteria	Firmicutes	Clostridia	Clostridiales			
ASV217	5215.868497	0.680716794	0.341964028	1.990609355	0.117925	0.204212	3.969	Bacteria	Firmicutes	Clostridia	Clostridiales	Ruminococcaceae	Oscillospira	
ASV119	3732.945236	2.462636262	0.892493602	2.759276097	0.128479	0.218862	1.732	Bacteria	Firmicutes	Clostridia	Clostridiales			
ASV158	862.8151294	-0.899903885	0.544615606	-1.652365219	0.129468	0.218862	10	Bacteria	Firmicutes	Clostridia	Clostridiales	Christensenellaceae		
ASV107	2904.832492	0.700370716	0.420996945	1.663600469	0.157097	0.259393	4.998	Bacteria	Firmicutes	Clostridia	Clostridiales			
ASV203	924.6405605	1.121870944	0.679298814	1.65151318	0.156377	0.259393	5.286	Bacteria	Firmicutes	Clostridia	Clostridiales			
ASV6	993.2789228	1.19955751	0.746473329	1.606966336	0.170274	0.274761	4.89	Bacteria	Bacteroidetes	Bacteroidia	Bacteroidales	S24-7		
ASV72	3533.021627	1.131958453	0.765208176	1.479281701	0.169862	0.274761	10	Bacteria	Firmicutes	Clostridia	Clostridiales	Ruminococcaceae		
ASV105	1380.055964	-0.285884604	0.174586194	-1.637498348	0.176929	0.282291	3.997	Bacteria	Firmicutes	Clostridia	Clostridiales			
ASV222	922.5774057	1.076122636	0.686615552	1.567285554	0.181937	0.283902	4.668	Bacteria	Firmicutes	Clostridia	Clostridiales	Ruminococcaceae	Oscillospira	
ASV283	1264.95673	-1.002150297	0.617410469	-1.623150801	0.18125	0.283902	3.924	Bacteria	Firmicutes	Clostridia	Clostridiales	Lachnospiraceae	[Ruminococcus]	gnavus
ASV88	710.5693328	0.964694095	0.623422925	1.547415174	0.190091	0.293402	4.411	Bacteria	Firmicutes	Clostridia	Clostridiales	Ruminococcaceae	Ruminococcus	
ASV75	1218.439386	0.985966548	0.669804037	1.472022404	0.199025	0.303888	5.179	Bacteria	Firmicutes	Clostridia	Clostridiales	Ruminococcaceae		
ASV225	1882.951989	1.42601294	0.962753696	1.481181476	0.209034	0.315775	4.222	Bacteria	Firmicutes	Clostridia	Clostridiales	Ruminococcaceae	Oscillospira	
ASV291	1082.907341	0.687273749	0.491445119	1.398475073	0.217971	0.325809	5.271	Bacteria	Firmicutes	Clostridia	Clostridiales	Lachnospiraceae		
ASV47	5486.33144	0.479229976	0.360413649	1.329666559	0.233222	0.344974	5.838	Bacteria	Firmicutes	Clostridia	Clostridiales	[Mogibacteriaceae]		
ASV59	652.0462247	0.718917481	0.494005533	1.455282246	0.254646	0.369367	2.603	Bacteria	Firmicutes	Clostridia	Clostridiales	Ruminococcaceae	Oscillospira	
ASV255	1509.878628	-1.306813956	1.0233994	-1.276934455	0.254916	0.369367	5.278	Bacteria	Firmicutes	Clostridia	Clostridiales			
ASV90	684.38679	0.747478238	0.581897673	1.284552718	0.26026	0.373303	4.565	Bacteria	Firmicutes	Clostridia	Clostridiales	Ruminococcaceae	Ruminococcus	
ASV179	949.9570801	-0.849044803	0.697549121	-1.217182815	0.273293	0.388077	5.485	Bacteria	Firmicutes	Clostridia	Clostridiales	Lachnospiraceae		
ASV13	13226.94926	-0.314242512	0.251398699	-1.249976682	0.279109	0.392411	4.022	Bacteria	Bacteroidetes	Bacteroidia	Bacteroidales	S24-7		
ASV148	1939.89934	0.947501135	0.791310818	1.197381753	0.293101	0.405015	4.29	Bacteria	Firmicutes	Clostridia	Clostridiales			
ASV232	631.093133	0.53533867	0.454972757	1.176638956	0.293778	0.405015	4.856	Bacteria	Firmicutes	Clostridia	Clostridiales	Ruminococcaceae	Oscillospira	
ASV233	685.0434934	0.748959271	0.692280256	1.081872934	0.304707	0.416043	10	Bacteria	Firmicutes	Clostridia	Clostridiales	Ruminococcaceae	Oscillospira	
ASV43	5191.220859	0.585448845	0.535905096	1.092448736	0.322228	0.433964	5.248	Bacteria	Firmicutes	Clostridia	Clostridiales	Dehalobacteriaceae	Dehalobacterium	
ASV170	611.566598	0.577994847	0.557110727	1.037486478	0.323945	0.433964	10	Bacteria	Firmicutes	Clostridia	Clostridiales	Lachnospiraceae	Coprococcus	
ASV103	633.7752112	0.53298449	0.469440064	1.135362171	0.331409	0.439814	3.324	Bacteria	Firmicutes	Clostridia	Clostridiales	Ruminococcaceae		
ASV24	59504.58457	-0.642975301	0.613822148	-1.047494462	0.341142	0.444423	5.195	Bacteria	Firmicutes	Bacilli	Turicibacterales	Turicibacteraceae	Turicibacter	
ASV114	6498.214844	-0.996941955	0.904998327	-1.101595357	0.340872	0.444423	3.483	Bacteria	Firmicutes	Clostridia	Clostridiales	Lachnospiraceae		
ASV231	15142.68118	-0.304335684	0.310086321	-0.981454724	0.349517	0.451194	10	Bacteria	Firmicutes	Clostridia	Clostridiales	Ruminococcaceae	Oscillospira	
ASV214	1077.267889	-0.562738092	0.599423502	-0.938798847	0.369955	0.473276	10	Bacteria	Firmicutes	Clostridia	Clostridiales	Ruminococcaceae	Ruminococcus	
ASV77	537.975352	0.384800466	0.431941666	0.890862114	0.393932	0.49945	10	Bacteria	Firmicutes	Clostridia	Clostridiales	Ruminococcaceae		
ASV150	6737.834218	-0.674593588	0.734497142	-0.918442767	0.400526	0.503316	5	Bacteria	Firmicutes	Clostridia	Clostridiales			
ASV51	533.980323	0.373568806	0.446276505	0.837079259	0.422102	0.525776	10	Bacteria	Firmicutes	Clostridia	Clostridiales			
ASV237	5908.69519	0.679079705	0.825362623	0.822765275	0.442803	0.546765	5.864	Bacteria	Firmicutes	Clostridia	Clostridiales	Clostridiaceae		
ASV154	1194.157647	-0.424643366	0.552650257	-0.768376311	0.460024	0.563133	10	Bacteria	Firmicutes	Clostridia	Clostridiales			
ASV290	543.596437	-0.386987864	0.563935724	-0.6862269	0.508168	0.616751	10	Bacteria	Firmicutes	Clostridia	Clostridiales	Lachnospiraceae	Dorea	
ASV71	10382.74306	0.126454471	0.195216233	0.64776617	0.531726	0.634496	10	Bacteria	Firmicutes	Clostridia	Clostridiales	Ruminococcaceae	Oscillospira	
ASV312	502.6590483	0.282484668	0.435214744	0.649069619	0.530917	0.634496	10	Bacteria	Firmicutes	Clostridia	Clostridiales	Lachnospiraceae		
ASV308	1268.098841	0.483953306	0.787266771	0.61472594	0.568719	0.672984	4.461	Bacteria	Firmicutes	Clostridia	Clostridiales			
ASV201	3307.664244	-0.407707617	0.784473219	-0.519721524	0.614567	0.721227	10	Bacteria	Firmicutes	Clostridia	Clostridiales	Lachnospiraceae		
ASV251	657.2312297	-0.244152127	0.488730371	-0.499564057	0.640036	0.74496	4.666	Bacteria	Firmicutes	Clostridia	Clostridiales			
ASV1	7344.862314	-0.338495397	0.707050479	-0.4787429	0.648879	0.746401	6.07	Bacteria	Proteobacteria	Betaproteobacteria	Burkholderiales	Alcaligeceae	Sutterella	
ASV140	718.3716437	-0.117817088	0.250119935	-0.471042376	0.661538	0.746401	4.108	Bacteria	Firmicutes	Clostridia	Clostridiales			
ASV198	1580.307586	0.284778748	0.597308495	0.476769961	0.656507	0.746401	4.348	Bacteria	Firmicutes	Clostridia	Clostridiales	Lachnospiraceae		
ASV319	1595.610573	-0.339188924	0.730583486	-0.464271271	0.6623	0.746401	4.913	Bacteria	Firmicutes	Clostridia	Clostridiales	Lachnospiraceae		
ASV280	7371.383978	0.363432266	0.827771863	0.439048828	0.669961	0.74909	10	Bacteria	Firmicutes	Clostridia	Clostridiales	Lachnospiraceae	[Ruminococcus]	gnavus
ASV5	17530.3107	-0.289752441	0.650076582	-0.445720473	0.675897	0.749823	4.622	Bacteria	Bacteroidetes	Bacteroidia	Bacteroidales	S24-7		
ASV186	577.9625438	-0.170612349	0.370244002	-0.460810569	0.682237	0.75099	2.482	Bacteria	Firmicutes	Clostridia	Clostridiales			
ASV274	619.4140918	-0.248458573	0.610130988	-0.407221692	0.692425	0.751807	10	Bacteria	Firmicutes	Clostridia	Clostridiales	Lachnospiraceae	[Ruminococcus]	gnavus
ASV277	594.5657361	-0.251938079	0.621128139	-0.405613694	0.693569	0.751807	10	Bacteria	Firmicutes	Clostridia	Clostridiales	Lachnospiraceae	[Ruminococcus]	gnavus
ASV55	1929.409749	-0.276359146	0.851580094	-0.324525136	0.752227	0.80313	10	Bacteria	Firmicutes	Clostridia	Clostridiales	Ruminococcaceae	Oscillospira	
ASV315	661.6529757	-0.201838328	0.578208178	-0.349075533	0.751568	0.80313	2.806	Bacteria	Firmicutes	Clostridia	Clostridiales	Lachnospiraceae		
ASV124	713.9204321	-0.155754098	0.728980934	-0.213660043	0.835105	0.884963	10	Bacteria	Firmicutes	Clostridia	Clostridiales			
ASV135	741.6760039	0.157112334	0.783908415	0.200421798	0.845168	0.888992	10	Bacteria	Firmicutes	Clostridia	Clostridiales			
ASV133	2701.110072	-0.134567104	0.817779086	-0.164551902	0.872575	0.911071	10	Bacteria	Firmicutes	Clostridia	Clostridiales			
ASV7	2944.590486	0.083459584	0.650858929	0.128229913	0.903677	0.936658	4.352	Bacteria	Bacteroidetes	Bacteroidia	Bacteroidales	S24-7		
ASV56	4532.993625	0.049685077	0.731333529	0.067937644	0.947174	0.956884	10	Bacteria	Firmicutes	Clostridia	Clostridiales	Ruminococcaceae	Oscillospira	
ASV215	4806.471588	-0.018794495	0.286861226	-0.065517726	0.950146	0.956884	5.337	Bacteria	Firmicutes	Clostridia	Clostridiales	Ruminococcaceae		
ASV226	12137.58874	0.028154331	0.416227721	0.067641652	0.948232	0.956884	6.105	Bacteria	Firmicutes	Clostridia	Clostridiales	Ruminococcaceae	Oscillospira	
ASV227	1090.200799	-0.040934489	0.572725306	-0.071473163	0.945644	0.956884	5.293	Bacteria	Firmicutes	Clostridia	Clostridiales	Ruminococcaceae		
ASV273	2329.881043	0.032749732	0.766237327	0.042740977	0.967684	0.967684	4.644	Bacteria	Firmicutes	Clostridia	Clostridiales	Lachnospiraceae	[Ruminococcus]	gnavus

Abbreviations: lfcSE, log2FoldChange standard error; padj, FDR corrected p-value; df, degrees of freedom.

Notes. Estimates were obtained from bias-corrected centered log2-ratio transformed regression as implemented in LinDA.

baseMean reflects the normalized average read count. Stat is the log2FoldChange / lfcSE.

Model degrees of freedom computed using the Satterthwaite approximation as implemented in lmerTest.

Supplemental Table S4. The difference in the relative abundance of genus-level phylotypes in male mice at Week 4 when compared to Week 0 in response to the HFD

Genus	baseMean	log2FoldChange	lfcSE	stat	pvalue	padj	reject	df
Lactococcus	5491.38442460	4.55486120	0.08867090	51.3681716	5.29E-08	1.22E-06	TRUE	5.0
Clostridium	2133.50856020	3.13027020	0.29633800	10.5631752	9.60E-07	1.10E-05	TRUE	10.0
Anaeroplasma	4168.37930850	-3.19225910	0.40810930	-7.8220697	1.43E-05	1.10E-04	TRUE	10.0
Akkermansia	195075.99045190	1.26809650	0.23833910	5.3205568	0.003139	0.018048	TRUE	5.0
Enterococcus	527.39528060	1.38349350	0.44034960	3.1418069	0.010478	0.042142	TRUE	10.0
Coprococcus	51683.11745110	-0.77813020	0.19764840	-3.9369426	0.010994	0.042142	TRUE	5.0
Ruminococcus	46453.71510560	-0.96652930	0.30122250	-3.2086889	0.023763	0.06832	FALSE	5.0
[Ruminococcus]	72126.82076120	-0.77367220	0.29018650	-2.666121	0.023649	0.06832	FALSE	10.0
Coprobacillus	417.43507180	1.03116080	0.42718020	2.4138779	0.036441	0.093127	FALSE	10.0
Desulfovibrio	244.64311870	0.22601010	0.08786500	2.5722422	0.049899	0.114769	FALSE	5.0
Blautia	276.34329210	0.40960940	0.18607480	2.201316	0.078963	0.165104	FALSE	5.0
Staphylococcus	287.96554600	0.47168680	0.27468230	1.7172083	0.146589	0.280962	FALSE	5.0
Butyricicoccus	460.87191480	-0.72830500	0.59477680	-1.2245013	0.248826	0.422064	FALSE	10.0
Dorea	2354.58664710	-0.70577650	0.55164650	-1.2794001	0.256909	0.422064	FALSE	5.0
Sutterella	1629.77456360	-0.39226720	0.43140020	-0.9092885	0.404896	0.590049	FALSE	5.0
Dehalobacterium	894.63583960	0.11556620	0.13142560	0.879328	0.41947	0.590049	FALSE	5.0
Adlercreutzia	7480.14960700	-0.11557830	0.13660640	-0.846068	0.436123	0.590049	FALSE	5.0
Roseburia	333.79253850	-0.24219580	0.42108030	-0.5751772	0.577883	0.738406	FALSE	10.0
Turicibacter	16249.41135590	0.33447830	0.63588770	0.5260021	0.621374	0.752189	FALSE	5.0
Lactobacillus	14679.22866100	-0.19319190	0.43674320	-0.4423467	0.676717	0.778225	FALSE	5.0
Anaerostipes	273.47593880	0.05812770	0.14929630	0.3893444	0.713052	0.780961	FALSE	5.0
Oscillospira	289908.86342410	0.05410890	0.26092290	0.2073752	0.843898	0.882257	FALSE	5.0
Bacteroides	286848.51113760	-0.00920230	0.17718000	-0.0519378	0.959601	0.959601	FALSE	10.0

Abbreviations: lfcSE, log2FoldChange standard error; padj, FDR corrected p-value; df, degrees of freedom.
Notes. Estimates were obtained from bias-corrected centered log2-ratio transformed regression as implemented in LinDA.
baseMean reflects the normalized average read count. Stat is the log2FoldChange / lfcSE.
Model degrees of freedom computed using the Satterthwaite approximation as implemented in lmerTest.

Supplemental Table S5. The difference in the relative abundance of amplicon sequence variants (ASV) in male mice at Week 4 when compared to Week 0 in response to the HFD

ASV	baseMean	log2FoldChange	lfcSE	stat	pvalue	padj	df	Kingdom	Phylum	Class	Order	Family	Genus	Species
ASV112	1618.631262	-3.328240472	0.161436979	-20.61634517	1.59E-09	2.26E-07	10.0	Bacteria	Firmicutes	Clostridia	Clostridiales			
ASV100	1746.414598	-3.442736635	0.187033512	-18.40705764	4.82E-09	3.42E-07	10.0	Bacteria	Firmicutes	Clostridia	Clostridiales	Ruminococcaceae	Ruminococcus	
ASV113	1924.402013	-3.588976613	0.262308167	-13.6822908	8.43E-08	3.99E-06	10.0	Bacteria	Firmicutes	Clostridia	Clostridiales			
ASV171	1853.054338	2.248433939	0.185183531	12.14165171	2.62E-07	9.29E-06	10.0	Bacteria	Firmicutes	Clostridia	Clostridiales	Lachnospiraceae	Coprococcus	
ASV134	8857.924455	4.003650139	0.369293757	10.84136966	7.54E-07	2.14E-05	10.0	Bacteria	Firmicutes	Clostridia	Clostridiales			
ASV95	1292.122671	1.705142298	0.168992047	10.09007421	1.46E-06	3.46E-05	10.0	Bacteria	Firmicutes	Clostridia	Clostridiales	Ruminococcaceae		
ASV104	4362.713571	-3.377591547	0.367306289	-9.195572327	3.41E-06	6.92E-05	10.0	Bacteria	Firmicutes	Clostridia	Clostridiales	Ruminococcaceae	Ruminococcus	
ASV294	2753.546545	-4.140855912	0.116893458	-35.42418861	5.62E-06	9.97E-05	3.8	Bacteria	Firmicutes	Clostridia	Clostridiales	Lachnospiraceae		
ASV61	3430.99881	3.146588093	0.200547247	15.69000894	1.29E-05	1.99E-04	5.3	Bacteria	Firmicutes	Clostridia	Clostridiales	Ruminococcaceae	Oscillospira	
ASV205	4809.354345	3.655408976	0.238812287	15.30662023	1.40E-05	1.99E-04	5.3	Bacteria	Firmicutes	Clostridia	Clostridiales	Peptostreptococcaceae		
ASV41	4926.262509	3.691116824	0.242728411	15.20677702	1.82E-05	2.15E-04	5.1	Bacteria	Firmicutes	Bacilli	Lactobacillales	Streptococcaceae	Lactococcus	
ASV262	3361.433442	-4.405583104	0.310793209	-14.17528753	1.69E-05	2.15E-04	5.4	Bacteria	Firmicutes	Clostridia	Clostridiales			
ASV163	2730.959559	2.824612008	0.254889806	11.08169862	5.26E-05	4.98E-04	5.6	Bacteria	Firmicutes	Clostridia	Clostridiales			
ASV194	2353.716148	1.871114392	0.096691411	19.35140223	4.91E-05	4.98E-04	3.9	Bacteria	Firmicutes	Clostridia	Clostridiales	Lachnospiraceae		
ASV210	1789.112318	2.151684711	0.1467915	14.65810153	5.05E-05	4.98E-04	4.6	Bacteria	Firmicutes	Clostridia	Clostridiales	Ruminococcaceae	Ruminococcus	
ASV146	1613.543957	-3.323497048	0.553813998	-6.001106977	1.32E-04	0.0011	10.0	Bacteria	Firmicutes	Clostridia	Clostridiales			
ASV168	1001.695015	-2.60356668	0.285667965	-9.113960966	1.31E-04	0.0011	5.7	Bacteria	Firmicutes	Clostridia	Clostridiales	Lachnospiraceae	Coprococcus	
ASV92	808.6461546	-2.983344259	0.191831165	-15.55192694	1.44E-04	0.00111	3.8	Bacteria	Firmicutes	Clostridia	Clostridiales	Ruminococcaceae	Ruminococcus	
ASV323	1233.737284	-2.919082275	0.49358639	-5.914025056	1.48E-04	0.00111	10.0	Bacteria	Firmicutes	Clostridia	Clostridiales	Lachnospiraceae		
ASV19	6321.369436	-1.454031212	0.108371143	-13.41714379	1.77E-04	0.00117	4.0	Bacteria	Bacteroidetes	Bacteroidia	Bacteroidales	Rikenellaceae		
ASV129	1160.571767	-2.798369687	0.322885197	-8.666763658	1.86E-04	0.00117	5.6	Bacteria	Firmicutes	Clostridia	Clostridiales			
ASV258	3078.020674	2.301549135	0.40078258	5.742637659	1.87E-04	0.00117	10.0	Bacteria	Firmicutes	Erysipelotrichi	Erysipelotrichales	Erysipelotrichaceae	Clostridium	cocleatum
ASV281	1338.344372	-3.041717377	0.530631307	-5.73226143	1.90E-04	0.00117	10.0	Bacteria	Firmicutes	Clostridia	Clostridiales	Lachnospiraceae	[Ruminococcus]	gnavus
ASV193	1178.179102	-2.849649995	0.503610892	-5.658435992	2.10E-04	0.00124	10.0	Bacteria	Firmicutes	Clostridia	Clostridiales	Lachnospiraceae		
ASV182	1245.016306	-2.901341651	0.371695814	-7.805688267	3.04E-04	0.00173	5.7	Bacteria	Firmicutes	Clostridia	Clostridiales	Lachnospiraceae		
ASV266	2132.544737	-2.687440891	0.23315452	-11.52643701	4.23E-04	0.00231	3.8	Bacteria	Firmicutes	Clostridia	Clostridiales	Lachnospiraceae	Anaerostipes	
ASV147	8420.485958	-4.20168718	0.550315456	-7.635052101	4.43E-04	0.00232	5.4	Bacteria	Firmicutes	Clostridia	Clostridiales			
ASV296	2657.779608	2.027282258	0.396707782	5.110265914	4.57E-04	0.00232	10.0	Bacteria	Firmicutes	Clostridia	Clostridiales	Lachnospiraceae		
ASV309	6263.650945	2.544407984	0.522746471	4.867384334	6.54E-04	0.0032	10.0	Bacteria	Firmicutes	Clostridia	Clostridiales	Lachnospiraceae		
ASV21	37732.41144	4.026691593	0.621942159	6.474382758	7.44E-04	0.00352	5.8	Bacteria	Verrucomicrobia	Verrucomicrobiae	Verrucomicrobiales	Verrucomicrobiaceae	Akkermansia	muciniphila
ASV318	3353.232326	-2.482174252	0.376046127	-6.600717501	7.70E-04	0.00353	5.6	Bacteria	Firmicutes	Clostridia	Clostridiales	Lachnospiraceae		
ASV165	5295.004387	-2.801663216	0.612979038	-4.570569369	0.001025	0.00455	10.0	Bacteria	Firmicutes	Clostridia	Clostridiales	Lachnospiraceae	Coprococcus	
ASV27	10946.52817	-2.413218423	0.53561841	-4.505480729	0.001133	0.00488	10.0	Bacteria	Tenericutes	Mollicutes	Anaeroplasmatales	Anaeroplasmataceae	Anaeroplasma	
ASV80	1177.008629	-2.207822855	0.364331515	-6.059928286	0.001588	0.00663	5.2	Bacteria	Actinobacteria	Coriobacteriia	Coriobacteriales	Coriobacteriaceae	Adlercreutzia	
ASV136	3313.238415	-2.600655905	0.610289097	-4.261350759	0.001659	0.00673	10.0	Bacteria	Firmicutes	Clostridia	Clostridiales			
ASV223	6075.140659	2.176575508	0.518853313	4.194972748	0.001843	0.00727	10.0	Bacteria	Firmicutes	Clostridia	Clostridiales	Ruminococcaceae	Oscillospira	
ASV11	9137.623951	-2.409456862	0.337600335	-7.137009684	0.001968	0.00755	4.0	Bacteria	Bacteroidetes	Bacteroidia	Bacteroidales	S24-7		
ASV138	684.0113338	-1.977937093	0.356810584	-5.543381227	0.002231	0.00834	5.3	Bacteria	Firmicutes	Clostridia	Clostridiales			
ASV46	469.4693176	-1.463167328	0.389194861	-3.759472374	0.003725	0.01356	10.0	Bacteria	Firmicutes	Clostridia	Clostridiales	[Mogibacteriaceae]		
ASV192	1774.939416	-2.524188511	0.49153127	-5.135356921	0.004112	0.0146	4.8	Bacteria	Firmicutes	Clostridia	Clostridiales	Lachnospiraceae		
ASV195	612.3737212	-1.863596246	0.523987537	-3.556565973	0.005211	0.01792	10.0	Bacteria	Firmicutes	Clostridia	Clostridiales	Lachnospiraceae		
ASV219	2058.97341	2.407213663	0.678776368	3.546401696	0.0053	0.01792	10.0	Bacteria	Firmicutes	Clostridia	Clostridiales	Ruminococcaceae	Oscillospira	
ASV126	1795.334062	2.149459634	0.496118029	4.332556992	0.005506	0.01818	5.7	Bacteria	Firmicutes	Clostridia	Clostridiales			
ASV97	2495.510421	2.112765644	0.279323535	7.563865471	0.005787	0.01868	2.8	Bacteria	Firmicutes	Clostridia	Clostridiales	Ruminococcaceae		
ASV175	1919.946408	-2.657065412	0.609571079	-4.358909903	0.006897	0.02176	5.1	Bacteria	Firmicutes	Clostridia	Clostridiales	Lachnospiraceae		
ASV216	16050.9536	-0.875866987	0.196653359	-4.453862334	0.00719	0.0222	4.8	Bacteria	Firmicutes	Clostridia	Clostridiales	Ruminococcaceae	Oscillospira	
ASV78	16489.23769	2.485496796	0.603026056	4.121707134	0.007646	0.02262	5.4	Bacteria	Firmicutes	Clostridia	Clostridiales	Ruminococcaceae		
ASV228	1487.350807	1.910270038	0.486387694	3.927463753	0.007585	0.02262	6.1	Bacteria	Firmicutes	Clostridia	Clostridiales	Ruminococcaceae	Oscillospira	
ASV284	8656.932758	-2.067263289	0.488849672	-4.22883231	0.008835	0.0256	4.8	Bacteria	Firmicutes	Clostridia	Clostridiales	Lachnospiraceae	[Ruminococcus]	gnavus
ASV189	2197.499129	-1.959597414	0.615810451	-3.182143808	0.009784	0.02779	10.0	Bacteria	Firmicutes	Clostridia	Clostridiales	Lachnospiraceae		
ASV261	699.0221969	-2.023092387	0.543413748	-3.72293192	0.011573	0.03222	5.5	Bacteria	Firmicutes	Clostridia	Clostridiales			
ASV321	708.9495803	-2.084261247	0.693289911	-3.006334313	0.0132	0.03605	10.0	Bacteria	Firmicutes	Clostridia	Clostridiales	Lachnospiraceae		
ASV76	2063.190528	-2.49793382	0.844084874	-2.959339632	0.014304	0.03832	10.0	Bacteria	Firmicutes	Clostridia	Clostridiales			
ASV70	533.5901995	-1.749685529	0.435814145	-4.014751588	0.01493	0.03855	4.1	Bacteria	Firmicutes	Clostridia	Clostridiales	Ruminococcaceae	Oscillospira	
ASV190	618.7562005	-2.02954177	0.526900893	-3.851847279	0.014822	0.03855	4.5	Bacteria	Firmicutes	Clostridia	Clostridiales	Lachnospiraceae		
ASV39	3373.695713	-1.965842242	0.528623426	-3.71879517	0.015644	0.03967	4.7	Bacteria	Firmicutes	Bacilli	Lactobacillales	Lactobacillaceae	Lactobacillus	
ASV211	658.5563661	-1.911043264	0.504931085	-3.784760575	0.016766	0.04177	4.3	Bacteria	Firmicutes	Clostridia	Clostridiales	Ruminococcaceae	Ruminococcus	
ASV292	743.2140061	-1.666686038	0.583892689	-2.854438955	0.017119	0.04191	10.0	Bacteria	Firmicutes	Clostridia	Clostridiales			
ASV181	587.8837049	-1.875647964	0.503744085	-3.723414366	0.017989	0.0433	4.3	Bacteria	Firmicutes	Clostridia	Clostridiales	Lachnospiraceae		
ASV173	654.7532192	-1.887023924	0.499222111	-3.779928577	0.019793	0.04684	4.0	Bacteria	Firmicutes	Clostridia	Clostridiales	Lachnospiraceae		
ASV84	4295.707975	-1.544742633	0.419533245	-3.682050595	0.023368	0.0544	3.8	Bacteria	Actinobacteria	Coriobacteriia	Coriobacteriales	Coriobacteriaceae	Adlercreutzia	
ASV127	2232.172766	1.740274962	0.583158834	2.984221212	0.027661	0.06235	5.4	Bacteria	Firmicutes	Clostridia	Clostridiales			
ASV252	996.1166779	-1.508905689	0.472862817	-3.191000934	0.027335	0.06235	4.6	Bacteria	Firmicutes	Clostridia	Clostridiales			
ASV268	1316.689955	-2.2080118	0.734857611	-3.004679775	0.029221	0.06484	5.1	Bacteria	Firmicutes	Clostridia	Clostridiales	Lachnospiraceae	[Ruminococcus]	gnavus
ASV153	1492.532945	1.895737328	0.176079932	10.76634518	0.035189	0.07687	1.2	Bacteria	Firmicutes	Clostridia	Clostridiales	Lachnospiraceae		
ASV298	7597.124174	-2.35981757	0.99419537	-2.373595412	0.039037	0.08399	10.0	Bacteria	Firmicutes	Clostridia	Clostridiales			
ASV18	9259.900056	-1.43908734	0.487775464	-2.950306945	0.040772	0.08641	4.1	Bacteria	Bacteroidetes	Bacteroidia	Bacteroidales	S24-7		
ASV109	1546.61387	-1.37429102	0.498574967	-2.756438071	0.042639	0.08904	4.7	Bacteria	Firmicutes	Clostridia	Clostridiales			
ASV130	2020.72692	2.368857063	0.929036197	2.549800612	0.045271	0.09317	5.7	Bacteria	Firmicutes	Clostridia	Clostridiales			
ASV279	461.6838675	-0.949271247	0.425083487	-2.233140725	0.049578	0.10057	10.0	Bacteria	Firmicutes	Clostridia	Clostridiales	Lachnospiraceae		
ASV143	1105.230815	-1.365745227	0.542396143	-2.517984767	0.050994	0.10199	5.3	Bacteria	Firmicutes	Clostridia	Clostridiales			
ASV128	6636.337593	1.352861467	0.613675965	2.204520862	0.052041	0.10264	10.0	Bacteria	Firmicutes	Clostridia	Clostridiales			
ASV40	82816.3222	-1.100496021	0.441885199	-2.49045685	0.058871	0.11403	4.6	Bacteria	Firmicutes	Bacilli	Lactobacillales	Lactobacillaceae	Lactobacillus	salivarius
ASV183	4575.301988	-1.561956108	0.734697181	-2.125986254	0.059422	0.11403	10.0	Bacteria	Firmicutes	Clostridia	Clostridiales	Lachnospiraceae		
ASV141	3096.738132	1.924287472	0.868713073	2.215101318	0.06762	0.12803	6.1	Bacteria	Firmicutes	Clostridia	Clostridiales			
ASV142	8071.171866	1.410641588	0.636042945	2.21784016	0.069825	0.13046	5.8	Bacteria	Firmicutes	Clostridia	Clostridiales			
ASV3	344579.9721	-0.593759613	0.27136605	-2.188039415	0.075407	0.13906	5.5	Bacteria	Bacteroidetes	Bacteroidia	Bacteroidales	Bacteroidaceae	Bacteroides	
ASV63	39699.78599	0.590592206	0.312277359	1.891242474	0.087882	0.15999	10.0	Bacteria	Firmicutes	Clostridia	Clostridiales	Ruminococcaceae	Oscillospira	
ASV73	903.0851932	-1.202593266	0.66675818	-1.803642312	0.101448	0.18007	10.0	Bacteria	Firmicutes	Clostridia	Clostridiales	Ruminococcaceae	Butyricicoccus	pullicaecorum
ASV208	561.8686368	-1.053792629	0.583558774	-1.805803763	0.101092	0.18007	10.0	Bacteria	Firmicutes	Clostridia	Clostridiales	Ruminococcaceae	Ruminococcus	
ASV116	954.6751609	1.249064534	0.704380446	1.773281103	0.106583	0.18685	10.0	Bacteria	Firmicutes	Clostridia	Clostridiales			
ASV217	5215.868497	0.680716794	0.341964028	1.990609355	0.117925	0.20421	4.0	Bacteria	Firmicutes	Clostridia	Clostridiales	Ruminococcaceae	Oscillospira	
ASV119	3732.945236	2.462636262	0.892493602	2.759276097	0.128479	0.21886	1.7	Bacteria	Firmicutes	Clostridia	Clostridiales			
ASV158	862.8151294	-0.899903885	0.544615606	-1.652365219	0.129468	0.21886	10.0	Bacteria	Firmicutes	Clostridia	Clostridiales	Christensenellaceae		
ASV107	2904.832492	0.700370716	0.420996945	1.663600469	0.157097	0.25939	5.0	Bacteria	Firmicutes	Clostridia	Clostridiales			
ASV203	924.6405605	1.121870944	0.679298814	1.65151318	0.156377	0.25939	5.3	Bacteria	Firmicutes	Clostridia	Clostridiales			
ASV6	993.2789228	1.19955751	0.746473329	1.606966336	0.170274	0.27476	4.9	Bacteria	Bacteroidetes	Bacteroidia	Bacteroidales	S24-7		
ASV72	3533.021627	1.131958453	0.765208176	1.479281701	0.169862	0.27476	10.0	Bacteria	Firmicutes	Clostridia	Clostridiales	Ruminococcaceae		
ASV105	1380.055964	-0.285884604	0.174586194	-1.637498348	0.176929	0.28229	4.0	Bacteria	Firmicutes	Clostridia	Clostridiales			
ASV222	922.5774057	1.076122636	0.686615552	1.567285554	0.181937	0.2839	4.7	Bacteria	Firmicutes	Clostridia	Clostridiales	Ruminococcaceae	Oscillospira	
ASV283	1264.95673	-1.002150297	0.617410469	-1.623150801	0.18125	0.2839	3.9	Bacteria	Firmicutes	Clostridia	Clostridiales	Lachnospiraceae	[Ruminococcus]	gnavus
ASV88	710.5693328	0.964694095	0.623422925	1.547415174	0.190091	0.2934	4.4	Bacteria	Firmicutes	Clostridia	Clostridiales	Ruminococcaceae	Ruminococcus	
ASV75	1218.439386	0.985966548	0.669804037	1.472022404	0.199025	0.30389	5.2	Bacteria	Firmicutes	Clostridia	Clostridiales	Ruminococcaceae		
ASV225	1882.951989	1.42601294	0.962753696	1.481181476	0.209034	0.31577	4.2	Bacteria	Firmicutes	Clostridia	Clostridiales	Ruminococcaceae	Oscillospira	
ASV291	1082.907341	0.687273749	0.491445119	1.398475073	0.217971	0.32581	5.3	Bacteria	Firmicutes	Clostridia	Clostridiales	Lachnospiraceae		
ASV47	5486.33144	0.479229976	0.360413649	1.329666559	0.233222	0.34497	5.8	Bacteria	Firmicutes	Clostridia	Clostridiales	[Mogibacteriaceae]		
ASV59	652.0462247	0.718917481	0.494005533	1.455282246	0.254646	0.36937	2.6	Bacteria	Firmicutes	Clostridia	Clostridiales	Ruminococcaceae	Oscillospira	
ASV255	1509.878628	-1.306813956	1.0233994	-1.276934455	0.254916	0.36937	5.3	Bacteria	Firmicutes	Clostridia	Clostridiales			
ASV90	684.38679	0.747478238	0.581897673	1.284552718	0.26026	0.3733	4.6	Bacteria	Firmicutes	Clostridia	Clostridiales	Ruminococcaceae	Ruminococcus	
ASV179	949.9570801	-0.849044803	0.697549121	-1.217182815	0.273293	0.38808	5.5	Bacteria	Firmicutes	Clostridia	Clostridiales	Lachnospiraceae		
ASV13	13226.94926	-0.314242512	0.251398699	-1.249976682	0.279109	0.39241	4.0	Bacteria	Bacteroidetes	Bacteroidia	Bacteroidales	S24-7		
ASV148	1939.89934	0.947501135	0.791310818	1.197381753	0.293101	0.40501	4.3	Bacteria	Firmicutes	Clostridia	Clostridiales			
ASV232	631.093133	0.53533867	0.454972757	1.176638956	0.293778	0.40501	4.9	Bacteria	Firmicutes	Clostridia	Clostridiales	Ruminococcaceae	Oscillospira	
ASV233	685.0434934	0.748959271	0.692280256	1.081872934	0.304707	0.41604	10.0	Bacteria	Firmicutes	Clostridia	Clostridiales	Ruminococcaceae	Oscillospira	
ASV43	5191.220859	0.585448845	0.535905096	1.092448736	0.322228	0.43396	5.2	Bacteria	Firmicutes	Clostridia	Clostridiales	Dehalobacteriaceae	Dehalobacterium	
ASV170	611.566598	0.577994847	0.557110727	1.037486478	0.323945	0.43396	10.0	Bacteria	Firmicutes	Clostridia	Clostridiales	Lachnospiraceae	Coprococcus	
ASV103	633.7752112	0.53298449	0.469440064	1.135362171	0.331409	0.43981	3.3	Bacteria	Firmicutes	Clostridia	Clostridiales	Ruminococcaceae		
ASV24	59504.58457	-0.642975301	0.613822148	-1.047494462	0.341142	0.44442	5.2	Bacteria	Firmicutes	Bacilli	Turicibacterales	Turicibacteraceae	Turicibacter	
ASV114	6498.214844	-0.996941955	0.904998327	-1.101595357	0.340872	0.44442	3.5	Bacteria	Firmicutes	Clostridia	Clostridiales	Lachnospiraceae		
ASV231	15142.68118	-0.304335684	0.310086321	-0.981454724	0.349517	0.45119	10.0	Bacteria	Firmicutes	Clostridia	Clostridiales	Ruminococcaceae	Oscillospira	
ASV214	1077.267889	-0.562738092	0.599423502	-0.938798847	0.369955	0.47328	10.0	Bacteria	Firmicutes	Clostridia	Clostridiales	Ruminococcaceae	Ruminococcus	
ASV77	537.975352	0.384800466	0.431941666	0.890862114	0.393932	0.49945	10.0	Bacteria	Firmicutes	Clostridia	Clostridiales	Ruminococcaceae		
ASV150	6737.834218	-0.674593588	0.734497142	-0.918442767	0.400526	0.50332	5.0	Bacteria	Firmicutes	Clostridia	Clostridiales			
ASV51	533.980323	0.373568806	0.446276505	0.837079259	0.422102	0.52578	10.0	Bacteria	Firmicutes	Clostridia	Clostridiales			
ASV237	5908.69519	0.679079705	0.825362623	0.822765275	0.442803	0.54677	5.9	Bacteria	Firmicutes	Clostridia	Clostridiales	Clostridiaceae		
ASV154	1194.157647	-0.424643366	0.552650257	-0.768376311	0.460024	0.56313	10.0	Bacteria	Firmicutes	Clostridia	Clostridiales			
ASV290	543.596437	-0.386987864	0.563935724	-0.6862269	0.508168	0.61675	10.0	Bacteria	Firmicutes	Clostridia	Clostridiales	Lachnospiraceae	Dorea	
ASV71	10382.74306	0.126454471	0.195216233	0.64776617	0.531726	0.6345	10.0	Bacteria	Firmicutes	Clostridia	Clostridiales	Ruminococcaceae	Oscillospira	
ASV312	502.6590483	0.282484668	0.435214744	0.649069619	0.530917	0.6345	10.0	Bacteria	Firmicutes	Clostridia	Clostridiales	Lachnospiraceae		
ASV308	1268.098841	0.483953306	0.787266771	0.61472594	0.568719	0.67298	4.5	Bacteria	Firmicutes	Clostridia	Clostridiales			
ASV201	3307.664244	-0.407707617	0.784473219	-0.519721524	0.614567	0.72123	10.0	Bacteria	Firmicutes	Clostridia	Clostridiales	Lachnospiraceae		
ASV251	657.2312297	-0.244152127	0.488730371	-0.499564057	0.640036	0.74496	4.7	Bacteria	Firmicutes	Clostridia	Clostridiales			
ASV1	7344.862314	-0.338495397	0.707050479	-0.4787429	0.648879	0.7464	6.1	Bacteria	Proteobacteria	Betaproteobacteria	Burkholderiales	Alcaligenaceae	Sutterella	
ASV140	718.3716437	-0.117817088	0.250119935	-0.471042376	0.661538	0.7464	4.1	Bacteria	Firmicutes	Clostridia	Clostridiales			
ASV198	1580.307586	0.284778748	0.597308495	0.476769961	0.656507	0.7464	4.3	Bacteria	Firmicutes	Clostridia	Clostridiales	Lachnospiraceae		
ASV319	1595.610573	-0.339188924	0.730583486	-0.464271271	0.6623	0.7464	4.9	Bacteria	Firmicutes	Clostridia	Clostridiales	Lachnospiraceae		
ASV280	7371.383978	0.363432266	0.827771863	0.439048828	0.669961	0.74909	10.0	Bacteria	Firmicutes	Clostridia	Clostridiales	Lachnospiraceae	[Ruminococcus]	gnavus
ASV5	17530.3107	-0.289752441	0.650076582	-0.445720473	0.675897	0.74982	4.6	Bacteria	Bacteroidetes	Bacteroidia	Bacteroidales	S24-7		
ASV186	577.9625438	-0.170612349	0.370244002	-0.460810569	0.682237	0.75099	2.5	Bacteria	Firmicutes	Clostridia	Clostridiales			
ASV274	619.4140918	-0.248458573	0.610130988	-0.407221692	0.692425	0.75181	10.0	Bacteria	Firmicutes	Clostridia	Clostridiales	Lachnospiraceae	[Ruminococcus]	gnavus
ASV277	594.5657361	-0.251938079	0.621128139	-0.405613694	0.693569	0.75181	10.0	Bacteria	Firmicutes	Clostridia	Clostridiales	Lachnospiraceae	[Ruminococcus]	gnavus
ASV55	1929.409749	-0.276359146	0.851580094	-0.324525136	0.752227	0.80313	10.0	Bacteria	Firmicutes	Clostridia	Clostridiales	Ruminococcaceae	Oscillospira	
ASV315	661.6529757	-0.201838328	0.578208178	-0.349075533	0.751568	0.80313	2.8	Bacteria	Firmicutes	Clostridia	Clostridiales	Lachnospiraceae		
ASV124	713.9204321	-0.155754098	0.728980934	-0.213660043	0.835105	0.88496	10.0	Bacteria	Firmicutes	Clostridia	Clostridiales			
ASV135	741.6760039	0.157112334	0.783908415	0.200421798	0.845168	0.88899	10.0	Bacteria	Firmicutes	Clostridia	Clostridiales			
ASV133	2701.110072	-0.134567104	0.817779086	-0.164551902	0.872575	0.91107	10.0	Bacteria	Firmicutes	Clostridia	Clostridiales			
ASV7	2944.590486	0.083459584	0.650858929	0.128229913	0.903677	0.93666	4.4	Bacteria	Bacteroidetes	Bacteroidia	Bacteroidales	S24-7		
ASV56	4532.993625	0.049685077	0.731333529	0.067937644	0.947174	0.95688	10.0	Bacteria	Firmicutes	Clostridia	Clostridiales	Ruminococcaceae	Oscillospira	
ASV215	4806.471588	-0.018794495	0.286861226	-0.065517726	0.950146	0.95688	5.3	Bacteria	Firmicutes	Clostridia	Clostridiales	Ruminococcaceae		
ASV226	12137.58874	0.028154331	0.416227721	0.067641652	0.948232	0.95688	6.1	Bacteria	Firmicutes	Clostridia	Clostridiales	Ruminococcaceae	Oscillospira	
ASV227	1090.200799	-0.040934489	0.572725306	-0.071473163	0.945644	0.95688	5.3	Bacteria	Firmicutes	Clostridia	Clostridiales	Ruminococcaceae		
ASV273	2329.881043	0.032749732	0.766237327	0.042740977	0.967684	0.96768	4.6	Bacteria	Firmicutes	Clostridia	Clostridiales	Lachnospiraceae	[Ruminococcus]	gnavus

Abbreviations: lfcSE, log2FoldChange standard error; padj, FDR corrected p-value; df, degrees of freedom.

Notes. Estimates were obtained from bias-corrected centered log2-ratio transformed regression as implemented in LinDA.

baseMean reflects the normalized average read count. Stat is the log2FoldChange / lfcSE.

Model degrees of freedom computed using the Satterthwaite approximation as implemented in lmerTest.

Supplemental Table S6. The difference in the relative abundance of genus-level phylotypes at Week 4 vs. Week 0 for female mice when compared to male mice in response to the HFD

Genus	baseMean	log2FoldChange	lfcSE	stat	pvalue	padj	reject	df
Anaerostipes	842.8964607	-1.188561803	0.133147093	-8.926682353	6.74E-06	1.55E-04	TRUE	9.4
Akkermansia	98358.59354	1.399241387	0.281689401	4.967319965	4.49E-04	0.004400	TRUE	10.8
Adlercreutzia	9847.894305	-0.479304832	0.09011064	-5.319070343	5.74E-04	0.004400	TRUE	8.5
Lactococcus	5739.419833	-0.322186431	0.086371549	-3.730237952	0.003660	0.021046	TRUE	10.4
Enterococcus	261.8002784	-0.755908729	0.267928466	-2.821308021	0.010548	0.048519	TRUE	20.0
Coprobacillus	227.5503865	-0.544833326	0.259858485	-2.096653972	0.048944	0.187619	FALSE	20.0
Anaeroplasma	6552.106551	0.735307637	0.346812374	2.120188588	0.057383	0.188543	FALSE	11.1
Oscillospira	280893.1121	0.313873717	0.155798764	2.014609802	0.072011	0.207032	FALSE	9.9
Blautia	175.6883915	-0.154680641	0.095457365	-1.620415988	0.133654	0.341561	FALSE	10.9
Staphylococcus	177.8437491	-0.173206779	0.129256262	-1.340026213	0.207220	0.408914	FALSE	11.0
Lactobacillus	42206.01208	-0.410304437	0.320365136	-1.280739979	0.230511	0.408914	FALSE	9.6
Dehalobacterium	2645.453738	0.281536058	0.196100395	1.435673083	0.183627	0.408914	FALSE	9.4
Clostridium	2775.089357	-0.284566533	0.230409053	-1.235049274	0.231125	0.408914	FALSE	20.0
Bacteroides	344755.5625	-0.18061336	0.150486723	-1.200194651	0.255770	0.420193	FALSE	10.8
Turicibacter	34938.72295	-0.412229116	0.424814293	-0.970374874	0.353572	0.542143	FALSE	10.6
Desulfovibrio	213.3877209	0.305653766	0.385248225	0.793394352	0.436860	0.627987	FALSE	20.0
Ruminococcus	49070.89278	0.097565645	0.176531245	0.552682022	0.591861	0.800753	FALSE	10.7
Coprococcus	39751.89935	0.056892259	0.132520821	0.429308076	0.676395	0.818794	FALSE	10.5
Dorea	1067.47391	0.231691815	0.53248929	0.43511075	0.672639	0.818794	FALSE	10.1
Sutterella	3754.901108	0.144152723	0.438269425	0.328913484	0.748444	0.860711	FALSE	10.9
[Ruminococcus]	74996.91209	0.050964589	0.204095595	0.249709402	0.808106	0.885068	FALSE	9.5
Roseburia	227.3909268	-0.035197707	0.3211398	-0.109602445	0.913817	0.955354	FALSE	20.0
Butyricicoccus	519.3959342	0.005291339	0.504813283	0.010481775	0.991741	0.991741	FALSE	20.0

Abbreviations: lfcSE, log2FoldChange standard error; padj, FDR corrected p-value; df, degrees of freedom.
Notes. Estimates were obtained from bias-corrected centered log2-ratio transformed regression as implemented in LinDA.
baseMean reflects the normalized average read count. Stat is the log2FoldChange / lfcSE.
Model degrees of freedom computed using the Satterthwaite approximation as implemented in lmerTest.

Supplemental Table S7. The difference in the relative abundance of amplicon sequence variants (ASV) at Week 4 vs. Week 0 for female mice when compared to male mice in response to the HFD

ASV	baseMean	log2FoldChange	lfcSE	stat	pvalue	padj	df	Kingdom	Phylum	Class	Order	Family	Genus	Species
ASV166	715.31497	1.78921	0.14484	12.35282	0	0	20.0	Bacteria	Firmicutes	Clostridia	Clostridiales	Lachnospiraceae	Coprococcus	
ASV294	687.85848	-2.11022	0.07171	-29.42627	0	0	10.0	Bacteria	Firmicutes	Clostridia	Clostridiales	Lachnospiraceae		
ASV185	866.37922	1.62817	0.07587	21.4587	0	0	8.5	Bacteria	Firmicutes	Clostridia	Clostridiales	Lachnospiraceae		
ASV266	899.88365	-1.20622	0.09891	-12.1953	0	0.00009	7.9	Bacteria	Firmicutes	Clostridia	Clostridiales	Lachnospiraceae	Anaerostipes	
ASV16	3037.21778	-0.56889	0.09917	-5.73683	0.00001	0.00041	20.0	Bacteria	Bacteroidetes	Bacteroidia	Bacteroidales	S24-7		
ASV129	493.15379	-1.18374	0.28796	-4.11079	0.00054	0.01354	20.0	Bacteria	Firmicutes	Clostridia	Clostridiales			
ASV153	987.75291	0.97321	0.19875	4.8967	0.00065	0.01354	9.9	Bacteria	Firmicutes	Clostridia	Clostridiales	Lachnospiraceae		
ASV156	569.77791	1.11501	0.2795	3.98934	0.00072	0.01354	20.0	Bacteria	Firmicutes	Clostridia	Clostridiales			
ASV175	554.67212	-1.38189	0.29385	-4.7027	0.00077	0.01354	10.3	Bacteria	Firmicutes	Clostridia	Clostridiales	Lachnospiraceae		
ASV195	1208.39308	1.21089	0.3135	3.86254	0.00097	0.01393	20.0	Bacteria	Firmicutes	Clostridia	Clostridiales	Lachnospiraceae		
ASV284	4902.68218	-1.73824	0.37805	-4.59792	0.00091	0.01393	10.3	Bacteria	Firmicutes	Clostridia	Clostridiales	Lachnospiraceae	[Ruminococcus]	gnavus
ASV296	4236.83928	1.4143	0.31484	4.49218	0.00119	0.01562	9.9	Bacteria	Firmicutes	Clostridia	Clostridiales	Lachnospiraceae		
ASV262	1124.09292	-1.65452	0.39039	-4.23813	0.00135	0.01645	11.2	Bacteria	Firmicutes	Clostridia	Clostridiales			
ASV194	2189.19683	1.26361	0.35408	3.56871	0.00192	0.0217	20.0	Bacteria	Firmicutes	Clostridia	Clostridiales	Lachnospiraceae		
ASV170	916.27486	-0.91179	0.26038	-3.50176	0.00225	0.02366	20.0	Bacteria	Firmicutes	Clostridia	Clostridiales	Lachnospiraceae	Coprococcus	
ASV268	943.94285	-1.82555	0.46122	-3.95811	0.00254	0.02505	10.3	Bacteria	Firmicutes	Clostridia	Clostridiales	Lachnospiraceae	[Ruminococcus]	gnavus
ASV21	91478.90329	1.27645	0.33766	3.7803	0.00298	0.02772	11.1	Bacteria	Verrucomicrobia	Verrucomicrobiae	Verrucomicrobiales	Verrucomicrobiaceae	Akkermansia	muciniphila
ASV272	895.04146	1.02896	0.31926	3.22292	0.00427	0.03745	20.0	Bacteria	Firmicutes	Clostridia	Clostridiales			
ASV76	773.51979	-1.21788	0.40895	-2.97804	0.00743	0.05804	20.0	Bacteria	Firmicutes	Clostridia	Clostridiales			
ASV181	1151.65079	1.14112	0.31458	3.62743	0.00722	0.05804	7.7	Bacteria	Firmicutes	Clostridia	Clostridiales	Lachnospiraceae		
ASV183	2207.9344	-1.36728	0.46167	-2.96158	0.00771	0.05804	20.0	Bacteria	Firmicutes	Clostridia	Clostridiales	Lachnospiraceae		
ASV41	5397.02449	-0.42341	0.13003	-3.25629	0.00889	0.06381	9.8	Bacteria	Firmicutes	Bacilli	Lactobacillales	Streptococcaceae	Lactococcus	
ASV48	380.79039	0.84009	0.30033	2.79724	0.01112	0.07642	20.0	Bacteria	Firmicutes	Clostridia	Clostridiales	[Mogibacteriaceae]		
ASV318	6685.54014	0.58664	0.19372	3.0283	0.01195	0.07866	10.6	Bacteria	Firmicutes	Clostridia	Clostridiales	Lachnospiraceae		
ASV216	10020.11306	-0.82386	0.2796	-2.9465	0.01366	0.08631	10.7	Bacteria	Firmicutes	Clostridia	Clostridiales	Ruminococcaceae	Oscillospira	
ASV15	2155.33273	-1.1305	0.42281	-2.6738	0.01459	0.08866	20.0	Bacteria	Bacteroidetes	Bacteroidia	Bacteroidales	S24-7		
ASV95	1256.07061	0.55499	0.20993	2.64364	0.01558	0.09117	20.0	Bacteria	Firmicutes	Clostridia	Clostridiales	Ruminococcaceae		
ASV7	3002.69912	1.26121	0.43586	2.89364	0.01728	0.09751	9.3	Bacteria	Bacteroidetes	Bacteroidia	Bacteroidales	S24-7		
ASV145	475.91081	0.72696	0.2956	2.4593	0.02316	0.12616	20.0	Bacteria	Firmicutes	Clostridia	Clostridiales			
ASV321	431.98441	-1.10492	0.41114	-2.68744	0.02426	0.12775	9.3	Bacteria	Firmicutes	Clostridia	Clostridiales	Lachnospiraceae		
ASV84	3509.36124	-1.10308	0.42521	-2.59421	0.02777	0.13712	9.5	Bacteria	Actinobacteria	Coriobacteriia	Coriobacteriales	Coriobacteriaceae	Adlercreutzia	
ASV189	2104.7675	-0.94856	0.36845	-2.57445	0.02705	0.13712	10.3	Bacteria	Firmicutes	Clostridia	Clostridiales	Lachnospiraceae		
ASV112	899.28184	-0.89416	0.35923	-2.48909	0.02979	0.13972	11.2	Bacteria	Firmicutes	Clostridia	Clostridiales			
ASV298	1377.15088	-1.1386	0.48758	-2.33519	0.03007	0.13972	20.0	Bacteria	Firmicutes	Clostridia	Clostridiales			
ASV13	59301.03713	-0.2248	0.08909	-2.52318	0.03261	0.14697	9.0	Bacteria	Bacteroidetes	Bacteroidia	Bacteroidales	S24-7		
ASV66	534.7484	-0.37234	0.14819	-2.51266	0.03349	0.14697	8.9	Bacteria	Firmicutes	Clostridia	Clostridiales	Ruminococcaceae	Oscillospira	
ASV162	687.24905	0.76389	0.28056	2.72275	0.0354	0.15116	5.9	Bacteria	Firmicutes	Clostridia	Clostridiales			
ASV19	1430.94755	-0.96751	0.42833	-2.2588	0.05084	0.21138	8.8	Bacteria	Bacteroidetes	Bacteroidia	Bacteroidales	Rikenellaceae		
ASV228	1682.54895	-0.48015	0.22401	-2.14338	0.05584	0.2262	10.8	Bacteria	Firmicutes	Clostridia	Clostridiales	Ruminococcaceae	Oscillospira	
ASV214	994.64774	-0.65213	0.32739	-1.99189	0.06021	0.23785	20.0	Bacteria	Firmicutes	Clostridia	Clostridiales	Ruminococcaceae	Ruminococcus	
ASV40	6161.50334	-0.8101	0.38418	-2.10866	0.06237	0.24037	9.6	Bacteria	Firmicutes	Bacilli	Lactobacillales	Lactobacillaceae	Lactobacillus	salivarius
ASV93	904.49285	0.96236	0.49439	1.94655	0.06577	0.24714	20.0	Bacteria	Firmicutes	Clostridia	Clostridiales	Ruminococcaceae		
ASV147	2178.12172	-1.24754	0.61514	-2.02805	0.06726	0.24714	11.1	Bacteria	Firmicutes	Clostridia	Clostridiales			
ASV67	518.99238	0.59353	0.29429	2.01683	0.07208	0.25461	9.8	Bacteria	Firmicutes	Clostridia	Clostridiales	Ruminococcaceae	Oscillospira	
ASV130	1304.33739	0.95252	0.47836	1.99122	0.07337	0.25461	10.4	Bacteria	Firmicutes	Clostridia	Clostridiales			
ASV142	6534.89879	-0.76451	0.4057	-1.88441	0.07413	0.25461	20.0	Bacteria	Firmicutes	Clostridia	Clostridiales			
ASV105	1346.36408	-0.33457	0.16814	-1.98988	0.0778	0.26153	9.0	Bacteria	Firmicutes	Clostridia	Clostridiales			
ASV63	53483.58116	0.34077	0.1854	1.83799	0.08096	0.2636	20.0	Bacteria	Firmicutes	Clostridia	Clostridiales	Ruminococcaceae	Oscillospira	
ASV64	734.27249	-0.82907	0.45308	-1.82986	0.08222	0.2636	20.0	Bacteria	Firmicutes	Clostridia	Clostridiales	Ruminococcaceae	Oscillospira	
ASV182	713.40801	-0.72965	0.40043	-1.82217	0.08342	0.2636	20.0	Bacteria	Firmicutes	Clostridia	Clostridiales	Lachnospiraceae		
ASV72	2313.24045	-0.70261	0.3912	-1.79602	0.08761	0.27143	20.0	Bacteria	Firmicutes	Clostridia	Clostridiales	Ruminococcaceae		
ASV160	522.56952	-0.78419	0.44084	-1.77883	0.09047	0.2749	20.0	Bacteria	Firmicutes	Clostridia	Clostridiales	Lachnospiraceae		
ASV39	6471.18631	-0.75778	0.42553	-1.78078	0.10487	0.28243	10.1	Bacteria	Firmicutes	Bacilli	Lactobacillales	Lactobacillaceae	Lactobacillus	
ASV103	786.01725	-0.6347	0.35583	-1.78374	0.10696	0.28243	9.3	Bacteria	Firmicutes	Clostridia	Clostridiales	Ruminococcaceae		
ASV114	8981.12291	-0.92984	0.49375	-1.88323	0.09575	0.28243	8.2	Bacteria	Firmicutes	Clostridia	Clostridiales	Lachnospiraceae		
ASV140	631.32376	-0.45468	0.25566	-1.77847	0.10725	0.28243	9.5	Bacteria	Firmicutes	Clostridia	Clostridiales			
ASV158	660.93737	-0.58462	0.33795	-1.72993	0.09905	0.28243	20.0	Bacteria	Firmicutes	Clostridia	Clostridiales	Christensenellaceae		
ASV205	2521.1337	0.69249	0.41026	1.6879	0.10696	0.28243	20.0	Bacteria	Firmicutes	Clostridia	Clostridiales	Peptostreptococcaceae		
ASV238	607.38438	-0.62693	0.36014	-1.74079	0.09708	0.28243	20.0	Bacteria	Firmicutes	Clostridia	Clostridiales			
ASV263	386.72967	-0.30463	0.16878	-1.80489	0.10216	0.28243	9.7	Bacteria	Firmicutes	Clostridia	Clostridiales			
ASV3	321573.1696	-0.27804	0.17428	-1.59534	0.12632	0.30652	20.0	Bacteria	Bacteroidetes	Bacteroidia	Bacteroidales	Bacteroidaceae	Bacteroides	
ASV27	6622.72638	0.51718	0.32404	1.59604	0.12616	0.30652	20.0	Bacteria	Tenericutes	Mollicutes	Anaeroplasmatales	Anaeroplasmataceae	Anaeroplasma	
ASV33	378.73487	-0.56288	0.36315	-1.55	0.13682	0.30652	20.0	Bacteria	Tenericutes	Mollicutes	RF39			
ASV43	2339.19067	0.42823	0.26262	1.63061	0.13542	0.30652	9.6	Bacteria	Firmicutes	Clostridia	Clostridiales	Dehalobacteriaceae	Dehalobacterium	
ASV47	4497.52698	-0.39196	0.23335	-1.67973	0.12224	0.30652	10.6	Bacteria	Firmicutes	Clostridia	Clostridiales	[Mogibacteriaceae]		
ASV87	482.15116	0.50158	0.293	1.71189	0.13774	0.30652	6.0	Bacteria	Firmicutes	Clostridia	Clostridiales			
ASV121	527.20395	0.73645	0.46918	1.56966	0.13218	0.30652	20.0	Bacteria	Firmicutes	Clostridia	Clostridiales			
ASV134	14437.22191	0.50325	0.29654	1.69711	0.11918	0.30652	10.5	Bacteria	Firmicutes	Clostridia	Clostridiales			
ASV219	1386.59315	0.70795	0.43635	1.62243	0.1342	0.30652	10.5	Bacteria	Firmicutes	Clostridia	Clostridiales	Ruminococcaceae	Oscillospira	
ASV222	1206.42551	-0.65771	0.42241	-1.55706	0.13514	0.30652	20.0	Bacteria	Firmicutes	Clostridia	Clostridiales	Ruminococcaceae	Oscillospira	
ASV312	565.02232	-0.47513	0.30419	-1.56196	0.13398	0.30652	20.0	Bacteria	Firmicutes	Clostridia	Clostridiales	Lachnospiraceae		
ASV61	3251.54806	-0.19262	0.12141	-1.58663	0.14327	0.3144	10.1	Bacteria	Firmicutes	Clostridia	Clostridiales	Ruminococcaceae	Oscillospira	
ASV18	20872.08855	-0.4768	0.30223	-1.57763	0.14942	0.32339	8.9	Bacteria	Bacteroidetes	Bacteroidia	Bacteroidales	S24-7		
ASV223	11264.43077	0.46787	0.31657	1.47792	0.15501	0.33096	20.0	Bacteria	Firmicutes	Clostridia	Clostridiales	Ruminococcaceae	Oscillospira	
ASV217	4193.18752	1.11272	0.71457	1.55718	0.15967	0.33637	7.7	Bacteria	Firmicutes	Clostridia	Clostridiales	Ruminococcaceae	Oscillospira	
ASV80	686.43596	-0.47687	0.32316	-1.47566	0.16769	0.34424	11.2	Bacteria	Actinobacteria	Coriobacteriia	Coriobacteriales	Coriobacteriaceae	Adlercreutzia	
ASV119	1409.13769	1.00103	0.69934	1.43139	0.16776	0.34424	20.0	Bacteria	Firmicutes	Clostridia	Clostridiales			
ASV133	1733.63165	0.83806	0.57126	1.46703	0.1731	0.35064	10.0	Bacteria	Firmicutes	Clostridia	Clostridiales			
ASV192	791.89993	-0.70612	0.4922	-1.43462	0.17954	0.35908	10.9	Bacteria	Firmicutes	Clostridia	Clostridiales	Lachnospiraceae		
ASV225	823.41592	0.8948	0.65244	1.37146	0.18542	0.36621	20.0	Bacteria	Firmicutes	Clostridia	Clostridiales	Ruminococcaceae	Oscillospira	
ASV300	1159.50211	-0.87827	0.63066	-1.39262	0.19154	0.37361	10.9	Bacteria	Firmicutes	Clostridia	Clostridiales	Lachnospiraceae		
ASV193	785.46869	-0.61651	0.44835	-1.37506	0.19599	0.37763	11.2	Bacteria	Firmicutes	Clostridia	Clostridiales	Lachnospiraceae		
ASV109	1432.18704	0.5551	0.4089	1.35755	0.20368	0.38717	10.3	Bacteria	Firmicutes	Clostridia	Clostridiales			
ASV258	2597.81689	-0.36329	0.27783	-1.30761	0.20584	0.38717	20.0	Bacteria	Firmicutes	Erysipelotrichi	Erysipelotrichales	Erysipelotrichaceae	Clostridium	cocleatum
ASV126	1913.79745	-0.35708	0.26895	-1.32766	0.2117	0.38908	10.8	Bacteria	Firmicutes	Clostridia	Clostridiales			
ASV230	394.11889	0.43752	0.33917	1.28997	0.21178	0.38908	20.0	Bacteria	Firmicutes	Clostridia	Clostridiales	Ruminococcaceae	Oscillospira	
ASV136	2861.75363	-0.63277	0.48201	-1.31279	0.2163	0.39282	10.9	Bacteria	Firmicutes	Clostridia	Clostridiales			
ASV174	782.54332	0.57077	0.44309	1.28818	0.22398	0.40069	11.1	Bacteria	Firmicutes	Clostridia	Clostridiales	Lachnospiraceae		
ASV280	5044.97824	0.69666	0.54252	1.28414	0.22571	0.40069	10.9	Bacteria	Firmicutes	Clostridia	Clostridiales	Lachnospiraceae	[Ruminococcus]	gnavus
ASV196	1004.06626	0.60807	0.49471	1.22914	0.23328	0.40954	20.0	Bacteria	Firmicutes	Clostridia	Clostridiales	Lachnospiraceae		
ASV138	447.57224	-0.47522	0.37947	-1.25232	0.23645	0.41053	11.0	Bacteria	Firmicutes	Clostridia	Clostridiales			
ASV168	1141.07132	0.26114	0.22049	1.18434	0.25016	0.42963	20.0	Bacteria	Firmicutes	Clostridia	Clostridiales	Lachnospiraceae	Coprococcus	
ASV11	22088.00363	0.28214	0.24223	1.16478	0.27433	0.45151	8.9	Bacteria	Bacteroidetes	Bacteroidia	Bacteroidales	S24-7		
ASV24	32770.24847	-0.5405	0.46725	-1.15677	0.27316	0.45151	10.4	Bacteria	Firmicutes	Bacilli	Turicibacterales	Turicibacteraceae	Turicibacter	
ASV154	625.81092	-0.32806	0.28785	-1.1397	0.26788	0.45151	20.0	Bacteria	Firmicutes	Clostridia	Clostridiales			
ASV215	1960.45757	-0.34603	0.30622	-1.13001	0.27184	0.45151	20.0	Bacteria	Firmicutes	Clostridia	Clostridiales	Ruminococcaceae		
ASV137	1297.89381	0.35902	0.31662	1.13392	0.28463	0.46058	9.5	Bacteria	Firmicutes	Clostridia	Clostridiales			
ASV198	1631.04871	0.39073	0.34676	1.1268	0.28778	0.46058	9.4	Bacteria	Firmicutes	Clostridia	Clostridiales	Lachnospiraceae		
ASV251	415.63299	0.42192	0.37714	1.11873	0.28915	0.46058	10.1	Bacteria	Firmicutes	Clostridia	Clostridiales			
ASV309	4255.00393	0.499	0.46057	1.08343	0.29151	0.46058	20.0	Bacteria	Firmicutes	Clostridia	Clostridiales	Lachnospiraceae		
ASV211	802.49126	0.469	0.45003	1.04216	0.30977	0.48284	20.0	Bacteria	Firmicutes	Clostridia	Clostridiales	Ruminococcaceae	Ruminococcus	
ASV231	22781.19564	0.20496	0.19748	1.0379	0.3117	0.48284	20.0	Bacteria	Firmicutes	Clostridia	Clostridiales	Ruminococcaceae	Oscillospira	
ASV157	802.25134	0.62792	0.61989	1.01296	0.32318	0.49575	20.0	Bacteria	Firmicutes	Clostridia	Clostridiales	Lachnospiraceae		
ASV92	758.2746	-0.40282	0.40191	-1.00229	0.32818	0.49618	20.0	Bacteria	Firmicutes	Clostridia	Clostridiales	Ruminococcaceae	Ruminococcus	
ASV322	386.97929	-0.45713	0.4576	-0.99898	0.32974	0.49618	20.0	Bacteria	Firmicutes	Clostridia	Clostridiales	Lachnospiraceae		
ASV71	10600.78892	-0.17697	0.17534	-1.0093	0.33512	0.49951	10.7	Bacteria	Firmicutes	Clostridia	Clostridiales	Ruminococcaceae	Oscillospira	
ASV5	29446.5905	0.29756	0.30157	0.9867	0.34837	0.50039	9.5	Bacteria	Bacteroidetes	Bacteroidia	Bacteroidales	S24-7		
ASV104	4152.64355	0.15087	0.15609	0.96658	0.3453	0.50039	20.0	Bacteria	Firmicutes	Clostridia	Clostridiales	Ruminococcaceae	Ruminococcus	
ASV127	1142.37456	0.41647	0.42387	0.98255	0.34741	0.50039	10.8	Bacteria	Firmicutes	Clostridia	Clostridiales			
ASV227	1559.13801	0.41029	0.41272	0.9941	0.34262	0.50039	10.5	Bacteria	Firmicutes	Clostridia	Clostridiales	Ruminococcaceae		
ASV255	484.13583	-0.52443	0.55076	-0.95218	0.35237	0.50157	20.0	Bacteria	Firmicutes	Clostridia	Clostridiales			
ASV210	2994.72598	0.30774	0.32678	0.94175	0.35756	0.50441	20.0	Bacteria	Firmicutes	Clostridia	Clostridiales	Ruminococcaceae	Ruminococcus	
ASV283	1784.96671	0.4716	0.49978	0.94362	0.36848	0.51522	9.6	Bacteria	Firmicutes	Clostridia	Clostridiales	Lachnospiraceae	[Ruminococcus]	gnavus
ASV148	899.70761	0.54663	0.62457	0.87521	0.40328	0.55407	9.4	Bacteria	Firmicutes	Clostridia	Clostridiales			
ASV190	885.57278	0.32083	0.3639	0.88165	0.40224	0.55407	8.5	Bacteria	Firmicutes	Clostridia	Clostridiales	Lachnospiraceae		
ASV107	4284.50568	0.17783	0.21116	0.84217	0.41977	0.57091	9.8	Bacteria	Firmicutes	Clostridia	Clostridiales			
ASV141	4492.90828	-0.44406	0.5333	-0.83265	0.42276	0.57091	11.0	Bacteria	Firmicutes	Clostridia	Clostridiales			
ASV97	1870.22524	0.21072	0.26222	0.80359	0.44005	0.57556	10.2	Bacteria	Firmicutes	Clostridia	Clostridiales	Ruminococcaceae		
ASV116	1183.58225	-0.39814	0.49468	-0.80483	0.44078	0.57556	9.4	Bacteria	Firmicutes	Clostridia	Clostridiales			
ASV125	522.94286	-0.32982	0.41447	-0.79575	0.43552	0.57556	20.0	Bacteria	Firmicutes	Clostridia	Clostridiales			
ASV146	1845.07758	0.24252	0.29669	0.81742	0.43188	0.57556	10.5	Bacteria	Firmicutes	Clostridia	Clostridiales			
ASV171	2934.84822	-0.14971	0.19521	-0.76694	0.46048	0.59636	10.2	Bacteria	Firmicutes	Clostridia	Clostridiales	Lachnospiraceae	Coprococcus	
ASV291	1169.10523	-0.25829	0.37333	-0.69185	0.50394	0.64733	10.6	Bacteria	Firmicutes	Clostridia	Clostridiales	Lachnospiraceae		
ASV252	747.7225	0.28778	0.42328	0.67988	0.51161	0.6519	10.3	Bacteria	Firmicutes	Clostridia	Clostridiales			
ASV186	548.71588	0.32881	0.50209	0.65488	0.52001	0.65207	20.0	Bacteria	Firmicutes	Clostridia	Clostridiales			
ASV281	1096.83288	-0.25286	0.37839	-0.66824	0.51763	0.65207	11.1	Bacteria	Firmicutes	Clostridia	Clostridiales	Lachnospiraceae	[Ruminococcus]	gnavus
ASV81	481.55234	0.22571	0.34366	0.6568	0.5259	0.65426	10.2	Bacteria	Actinobacteria	Coriobacteriia	Coriobacteriales	Coriobacteriaceae	Adlercreutzia	
ASV128	7889.88211	-0.20468	0.3779	-0.54163	0.59406	0.73329	20.0	Bacteria	Firmicutes	Clostridia	Clostridiales			
ASV163	2372.76571	-0.07479	0.16026	-0.46668	0.64577	0.79095	20.0	Bacteria	Firmicutes	Clostridia	Clostridiales			
ASV220	1470.33653	0.25268	0.54805	0.46105	0.65492	0.79598	9.7	Bacteria	Firmicutes	Clostridia	Clostridiales	Ruminococcaceae	Oscillospira	
ASV319	682.91374	-0.16276	0.3691	-0.44098	0.66984	0.8079	8.8	Bacteria	Firmicutes	Clostridia	Clostridiales	Lachnospiraceae		
ASV226	17029.41118	0.0656	0.16257	0.4035	0.69443	0.83121	10.8	Bacteria	Firmicutes	Clostridia	Clostridiales	Ruminococcaceae	Oscillospira	
ASV100	1697.78968	-0.04241	0.11288	-0.3757	0.71435	0.84863	10.9	Bacteria	Firmicutes	Clostridia	Clostridiales	Ruminococcaceae	Ruminococcus	
ASV90	632.2405	-0.12808	0.36525	-0.35066	0.73299	0.85195	10.2	Bacteria	Firmicutes	Clostridia	Clostridiales	Ruminococcaceae	Ruminococcus	
ASV150	2033.58923	-0.20163	0.57616	-0.34996	0.73333	0.85195	10.4	Bacteria	Firmicutes	Clostridia	Clostridiales			
ASV178	481.77349	0.18048	0.49686	0.36323	0.72377	0.85195	10.3	Bacteria	Firmicutes	Clostridia	Clostridiales	Lachnospiraceae		
ASV237	3614.20011	0.21979	0.66499	0.33052	0.74716	0.86169	11.1	Bacteria	Firmicutes	Clostridia	Clostridiales	Clostridiaceae		
ASV55	1000.44614	-0.14392	0.48812	-0.29484	0.77427	0.86879	9.8	Bacteria	Firmicutes	Clostridia	Clostridiales	Ruminococcaceae	Oscillospira	
ASV73	650.98798	-0.13556	0.47274	-0.28676	0.77725	0.86879	20.0	Bacteria	Firmicutes	Clostridia	Clostridiales	Ruminococcaceae	Butyricicoccus	pullicaecorum
ASV77	453.851	-0.062	0.2182	-0.28416	0.78195	0.86879	10.3	Bacteria	Firmicutes	Clostridia	Clostridiales	Ruminococcaceae		
ASV88	437.57465	0.12351	0.45717	0.27016	0.7898	0.86879	20.0	Bacteria	Firmicutes	Clostridia	Clostridiales	Ruminococcaceae	Ruminococcus	
ASV113	1965.70431	0.04129	0.15862	0.26028	0.79731	0.86879	20.0	Bacteria	Firmicutes	Clostridia	Clostridiales			
ASV143	705.24807	0.11829	0.44741	0.2644	0.79641	0.86879	10.9	Bacteria	Firmicutes	Clostridia	Clostridiales			
ASV273	6983.61464	0.11021	0.40021	0.27538	0.78942	0.86879	8.8	Bacteria	Firmicutes	Clostridia	Clostridiales	Lachnospiraceae	[Ruminococcus]	gnavus
ASV290	492.8301	0.09801	0.35949	0.27264	0.78792	0.86879	20.0	Bacteria	Firmicutes	Clostridia	Clostridiales	Lachnospiraceae	Dorea	
ASV285	630.31268	0.07988	0.35459	0.22527	0.8261	0.894	10.5	Bacteria	Firmicutes	Clostridia	Clostridiales	Lachnospiraceae	Dorea	
ASV165	5714.30067	-0.08113	0.40904	-0.19835	0.84477	0.90701	20.0	Bacteria	Firmicutes	Clostridia	Clostridiales	Lachnospiraceae	Coprococcus	
ASV232	541.66246	-0.05197	0.2794	-0.18601	0.85617	0.90701	10.0	Bacteria	Firmicutes	Clostridia	Clostridiales	Ruminococcaceae	Oscillospira	
ASV308	2506.58196	0.07545	0.41949	0.17986	0.86108	0.90701	9.4	Bacteria	Firmicutes	Clostridia	Clostridiales			
ASV323	1142.84636	-0.07583	0.40296	-0.18818	0.85263	0.90701	20.0	Bacteria	Firmicutes	Clostridia	Clostridiales	Lachnospiraceae		
ASV59	523.84373	0.05768	0.37108	0.15545	0.87961	0.92038	9.9	Bacteria	Firmicutes	Clostridia	Clostridiales	Ruminococcaceae	Oscillospira	
ASV179	439.78636	-0.05572	0.42895	-0.1299	0.8991	0.93459	10.5	Bacteria	Firmicutes	Clostridia	Clostridiales	Lachnospiraceae		
ASV78	15953.78281	-0.03687	0.34495	-0.10687	0.91687	0.94683	10.7	Bacteria	Firmicutes	Clostridia	Clostridiales	Ruminococcaceae		
ASV1	3522.18687	0.03503	0.38404	0.09121	0.92903	0.95316	10.6	Bacteria	Proteobacteria	Betaproteobacteria	Burkholderiales	Alcaligenaceae	Sutterella	
ASV75	784.21766	-0.03169	0.38762	-0.08176	0.93643	0.95456	10.1	Bacteria	Firmicutes	Clostridia	Clostridiales	Ruminococcaceae		
ASV56	7174.82951	-0.01329	0.34052	-0.03902	0.96926	0.96926	20.0	Bacteria	Firmicutes	Clostridia	Clostridiales	Ruminococcaceae	Oscillospira	
ASV124	619.29049	0.013	0.26317	0.04941	0.96166	0.96926	9.1	Bacteria	Firmicutes	Clostridia	Clostridiales			
ASV201	4611.319	0.02124	0.50051	0.04244	0.96657	0.96926	20.0	Bacteria	Firmicutes	Clostridia	Clostridiales	Lachnospiraceae		

Abbreviations: lfcSE, log2FoldChange standard error; padj, FDR corrected p-value; df, degrees of freedom.

Notes. Estimates were obtained from bias-corrected centered log2-ratio transformed regression as implemented in LinDA.

baseMean reflects the normalized average read count. Stat is the log2FoldChange / lfcSE.

Model degrees of freedom computed using the Satterthwaite approximation as implemented in lmerTest.
